# Reductive Nitrogen Species Activation via Pulsed Electrolysis: Recent Advances and Future Prospects

**DOI:** 10.1002/anie.202516909

**Published:** 2025-10-24

**Authors:** Kiarash Torabi, Rongji Liu, David Leander Troglauer, Christean Nickel, Guillermo Corea, Tiansheng Bai, Deping Li, Lijie Ci, Bahareh Feizi Mohazzab, Dandan Gao

**Affiliations:** ^1^ Department of Chemistry Johannes Gutenberg University Mainz Duesbergweg 10–14 Mainz 55128 Germany; ^2^ State Key Laboratory of Advanced Welding and Joining School of Materials Science and Engineering Harbin Institute of Technology (Shenzhen) Shenzhen 518055 People's Republic of China

**Keywords:** C─N Bond coupling, Electrocatalyst modulation, Nitrogen activation, Pulsed electrolysis, Reaction microenvironment

## Abstract

The electrochemical reduction of nitrogen species offers a sustainable route to mitigate environmental nitrogen pollution while enabling the production of value‐added chemicals such as ammonia, hydroxylamine, and C─N coupled organonitrogen compounds. However, the practical implementation of conventional potentiostatic methods is hindered by poor product selectivity and competing hydrogen evolution. Pulsed electrolysis has emerged as a transformative strategy to address these challenges by synchronizing catalyst surface dynamics with local microenvironmental changes and reaction kinetics. This mini‐review highlights recent advances in pulsed electrolysis for nitrogen species reduction, with a particular focus on how dynamic potentials influence electrocatalyst behavior and the surrounding reaction environment. Key mechanistic insights and cutting‐edge research findings are discussed, followed by an outlook on established systems and future directions toward scalable and energy‐efficient nitrogen activation.

## Graphical abstract

Pulsed electrolysis enables selective nitrogen species reduction by dynamically tuning electrocatalyst surfaces and local reaction microenvironments. This minireview highlights advances in producing ammonia and C─N coupled organonitrogen compounds under pulsed electrolysis conditions. In addition, mechanistic insights and future directions are discussed. EDL = electric double layer.

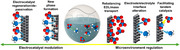

## 1 Introduction

The nitrogen cycle is a cornerstone of global ecosystems and human industries, yet its mismanagement poses escalating environmental and societal challenges.^[^ ^1^, ^2^, ^3^ ^]^ In particular, excessive nitrate (NO_3_^−^) and nitrite (NO_2_^−^) runoff from agriculture and industrial wastewater has led to eutrophication and toxic algal blooms.^[^ ^4^, ^5^ ^]^ In addition, the emission of nitrous oxide (N_2_O), a byproduct of industrial nitrogen fixation, also burdens significantly to climate change, with a global warming potential approximately 300 times that of CO_2_.^[^ ^6^, ^7^ ^]^ Meanwhile, nitrogen‐rich compounds, such as hydroxylamine (NH_2_OH),^[^ ^8^, ^9^ ^]^ ammonia (NH_3_),^[^ ^10^, ^11^ ^]^ and urea (CO(NH_2_)_2_)^[^ ^12^, ^13^ ^]^ are indispensable for fertilizers, energy storage, and industrial chemicals.

In this context, electrochemical nitrogen species reduction has emerged as a promising strategy for restoring the disrupted nitrogen cycle by “turning waste into treasure” under ambient conditions,^[^ ^14^ ^]^ particularly when driven by renewably “green” electricity sources. This approach offers a sustainable pathway for converting nitrogen‐based pollutants into value‐added products. When integrated with the reduction of suitable carbon sources (e.g., CO_2_, CO, ketones, or keto acids), it further enables C─N bond formation, thereby broadening the spectrum of valuable chemicals produced, such as amides, amines, amino acids, and oximes.^[^ ^5^, ^15^, ^16^, ^17^, ^18^ ^]^

Despite notable progress in recent years, conventional nitrogen species reduction is typically conducted under potentiostatic (constant potential) conditions, which present critical challenges related to catalyst stability and product selectivity.^[^ ^4^ ^]^ For instance, prolonged cathodic operation in either alkaline or acidic electrolytes can lead to electrode degradation, component agglomeration, and elemental dissolution, ultimately shortening catalyst lifespan.^[^ ^19^, ^20^ ^]^ Moreover, the complex proton‐coupled multielectron transfer pathways often result in the formation of multiple N‐containing intermediates and a mixture of products, thereby limiting selectivity (SE).^[^ ^21^ ^]^ These challenges are further compounded by the hydrogen evolution reaction (HER), which competes with nitrogen species reduction in aqueous media, particularly at more negative potentials, leading to decreased Faradaic efficiency (FE) and reduced yield rate (YR).^[^ ^22^ ^]^ Collectively, these limitations arise from static electrochemical conditions that cannot dynamically modulate catalyst evolution or steer reaction pathways, underscoring the urgent need for advanced engineering strategies.

To this end, pulsed electrolysis, a technique that periodically alternates voltage or current, offers a transformative synergy between catalyst design and electrochemical engineering.^[^ ^23^ ^]^ By cyclically modulating the applied potential, pulsed operation can prolong catalyst lifespan by dynamically restructuring catalyst surfaces, suppressing competing reactions such as HER, and enhancing the kinetics of intermediate adsorption/desorption.^[^ ^24^ ^]^ Moreover, pulsed electrolysis aligns with circular economy principles by facilitating energy‐efficient integration with renewable power sources.^[^ ^25^ ^]^ However, a comprehensive summary of the electrocatalytic mechanisms underpinning pulsed bias in the emerging field of reductive nitrogen species activation is currently absent.

This minireview explores how pulsed electrolysis functions as a critical interface between fundamental catalyst modulation, reaction microenvironment, and electrochemical engineering, thereby enabling scalable and sustainable approaches to managing the nitrogen cycle. First, we provide a concise overview of the fundamental principles of pulsed electrolysis in the nitrogen species reduction. Second, we discuss pioneering studies that elucidate mechanistic insights, followed by strategies for C─N bond formation. Finally, we highlight emerging research directions that, in the authors’ view, hold substantial potential to advance both fundamental understanding and application‐oriented development in this rapidly evolving field.

## 2 Fundamentals of Pulsed Electrolysis in Advancing Nitrogen Species Reduction

**Figure 1:**
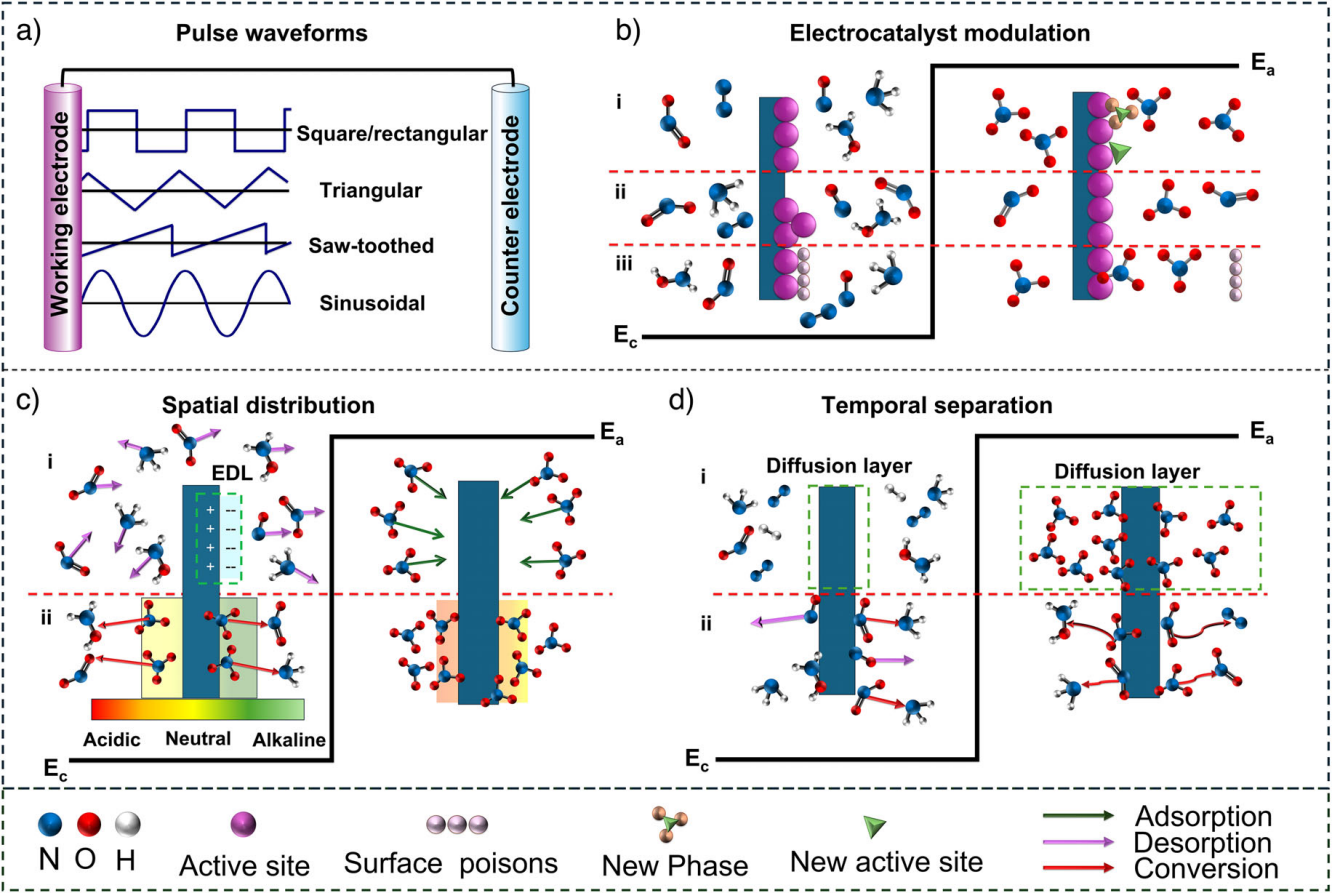
A schematic representation of a) various pulse shapes, b) electrocatalyst modulation, c) spatial distribution, and d) temporal separation.

Pulsed electrolysis strategy has the potential to regulate electrochemical performance, requiring optimization and fine‐tuning of pulsed parameters (Figure 1a), which are classified by two main categories: time‐ and potential‐dependent parameters.^[^ ^23^, ^25^ ^]^ Concretely, time‐dependent factors include the pulse period, frequency, and duty cycle, which determine the residence time in each state, mass transport and intermediate lifetimes, and the balance between conversion and relaxation.^[^ ^26^, ^27^, ^28^ ^]^ Potential‐dependent factors comprise amplitude, pulse shape (e.g., square/rectangular, triangular, saw‐toothed, sinusoidal, Figure 1a), and direction.^[^ ^29^, ^30^ ^]^ The amplitude, i.e., the potential difference between anodic and cathodic phases, sets the driving force for electron transfer, while pulse shape and direction dictate how potential changes modulate adsorbate behavior and interfacial dynamics.

In general, pulsed electrolysis enhances reaction rate and selectivity while beneficially reducing energy consumption.^[^ ^23^, ^31^, ^32^ ^]^ Particularly, this section lays focus on fundamentals of pulsed electrolysis in promoting nitrogen species reduction, specifically emphasizing electrocatalyst modulation (Figure 1b) and reaction microenvironment regulation via spatial distribution and temporal separation (Figure 1c,d). Throughout this review, the terms for anodic and cathodic potentials refer exclusively to the more positive and more negative voltages used in the pulsed electrolysis profile, respectively, without necessarily indicating a specific oxidation or reduction process.

**Electrocatalyst modulation** (Figure 1b): Pulsed potentials enable dynamic surface restructuring, which can expose fresh active sites or stabilize oxidation states that are transient under steady‐state conditions. For example, in cathodic duration, the intermediates are adsorbed on the surface, while anodic pulses can i) form new phases/active sites, ii) regenerate active sites, and iii) remove surface‐bound poisons (e.g., undesired intermediates accumulation) for de‐passivation, favoring adsorption of nitrogen species reactant in the next cathodic process.^[^ ^33^, ^34^ ^]^

**Spatial distribution** (Figure 1c): i) Modulation of the electric double layer (EDL) plays a crucial role in determining the local concentration of nitrogen species and protons for hydrogenation near the electrode surface. Applying off/rest time can discharge EDL,^[^ ^23^, ^35^ ^]^ whereas applying an anodic potential allows residual charge in the EDL to continue driving the partial reduction of adsorbed N‐containing intermediates while simultaneously promoting ion redistribution that prepares the interface for the next cathodic cycle.^[^ ^29^, ^36^ ^]^ This dynamic control over the interfacial environment enhances the efficiency of multielectron transfer steps required for complete nitrogen reduction. ii) pH interface alteration is of utmost importance since the local pH near the electrode surface significantly influences the reduction pathway of nitrogen species.^[^ ^37^ ^]^ As a prime example, nitrate reduction to ammonia is favored under mildly acidic to neutral conditions, while excessive alkalinity can hinder proton availability.^[^ ^38^ ^]^ It offers another powerful lever to steer the system toward desired products with improved efficiency and selectivity compared to conventional steady‐state electrolysis.

**Temporal separation** (Figure 1d): i) Rebalancing mass transport is key for nitrogen species reduction to avoid local depletion of reactants near the electrode surface, especially at high current densities. In this context, pulsed electrolysis can significantly mitigate the adverse concentration gradient that limits reaction rates by allowing the anodic period to refresh the diffusion layer and by restoring the supply of nitrogen species to the catalyst surface.^[^ ^25^ ^]^ This effect is valuable for removing entrapped bubbles (e.g., H_2_ or N_2_) near the catalyst surface, which impedes species diffusion. ii) Separation of tandem reaction steps enables dynamic manipulation of surface adsorption due to precise control of potential and timing under pulsed electrolysis. In this regard, reactive intermediates are formed during cathodic time, which will be reorganized, stabilized, or desorbed during anodic time, preventing over‐reduction or undesired side reactions.^[^ ^29^, ^39^ ^]^

## 3 Reductive Nitrogen Species Activation: Mechanisms and Systems

### 3.1 Electrocatalyst Modulation

Electrocatalyst modulation during pulsed electrolysis plays a pivotal role in enhancing catalytic performance by dynamically altering the catalyst surface and its local chemical environment. The application of alternating anodic and cathodic potentials induces various modifications to the electrocatalyst, which can be broadly classified into three categories: new active phase formation, electrocatalyst regeneration, and electrocatalyst de‐passivation.

#### 3.1.1 New Active Phase Formation

The in situ formation of a new active phase is often observed during pulsed electrolysis, possessing superior electronic structures and binding properties, thereby promoting more efficient intermediate adsorption and selective conversion. In this context, Cu‐based catalysts, especially oxide‐derived Cu catalysts^[^ ^40^, ^41^, ^42^, ^43^, ^44^ ^]^ and Cu oxide‐based composite catalysts,^[^ ^45^, ^46^, ^47^, ^48^, ^49^, ^50^ ^]^ have been widely explored.

**Figure 2:**
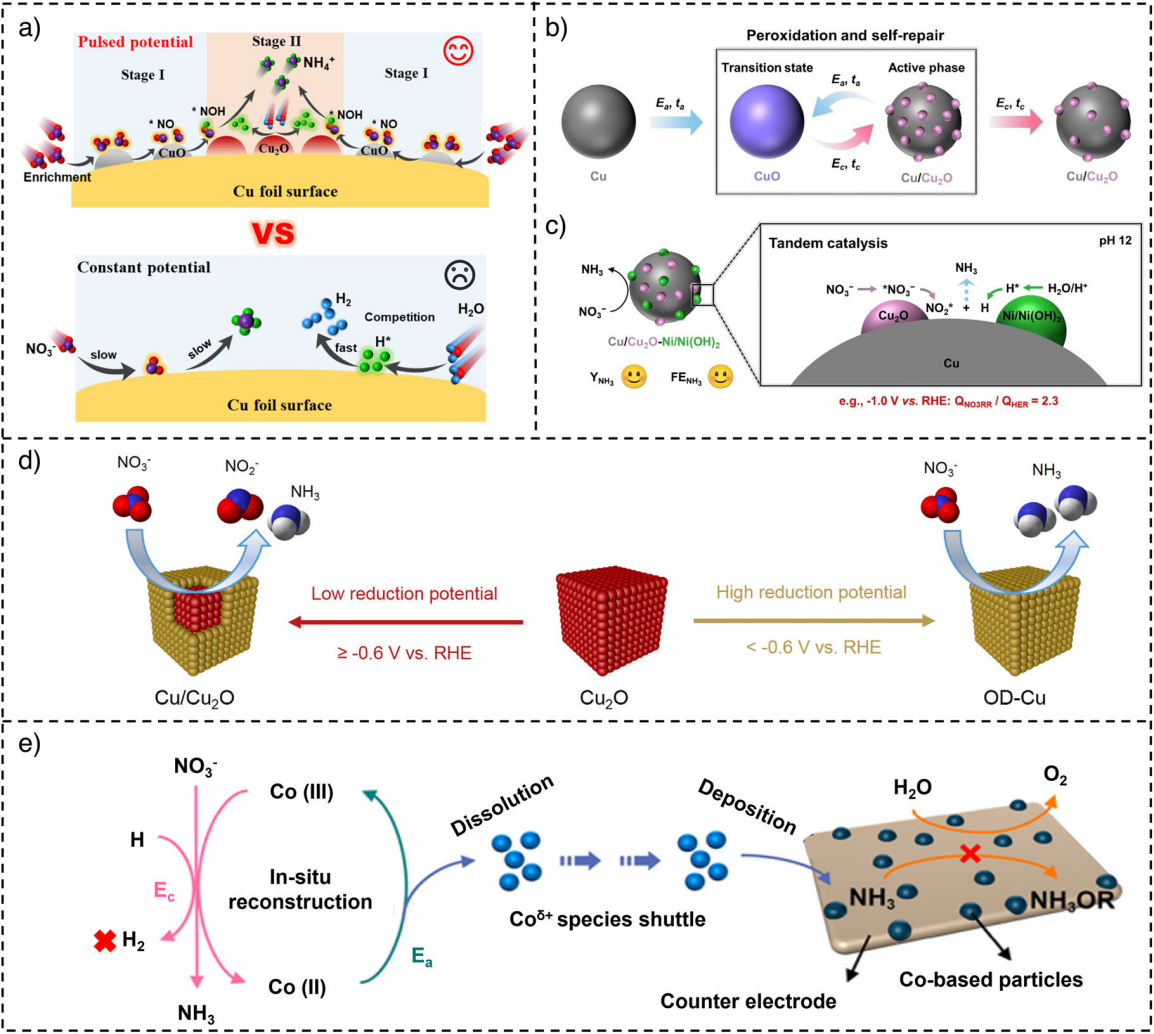
An illustration of a) pulsed and static electrolysis of NO_3_^−^ to NH_4_^+^ on Cu foil (reproduced with permission from Ref. [51] Copyright © 2025 Elsevier), b) in situ electrochemical peroxidation and self‐repair of Cu under pulsed NO_3_^−^RR at pH 14, c) tandem reaction scheme for Cu_50_Ni_50_ under pulsed NO_3_^−^RR at pH 12 (reproduced with permission from Ref. [52] Copyright © 2023 Wiley‐VCH), d) potential‐driven electrochemical restructuring of Cu_2_O cubes (reproduced with permission from Ref. [53] Copyright © 2024 American Chemical Society (ACS)), and e) the pulsed NO_3_^−^RR mechanism on Co‐based catalysts (adopted with permission from Ref. [61] Copyright © 2024 ACS).

In one study, the in situ formation of CuO/Cu_2_O interface is observed on the Cu foil cathode employed for enhanced NO_3_^−^RR under pulsed electrolysis.^[^ ^51^ ^]^ Concretely, CuO enriches NO_3_^−^ adsorption and initiates the reduction of NO_3_^−^ to NO_2_^−^, while Cu_2_O contributes to the hydrogenation of NO_2_^−^ to NH_3_ (Figure 2a). Pulsed electrolysis notably enhanced NO_3_^−^ removal from ∼5% to 95% within 3 h and improved NH_4_⁺ SE from 72% to 95%. In a related work, the formation of an active Cu/Cu_2_O heterojunction structure (Figure 2b) is also observed on the Cu nanocrystal deposited on Cu foil under pulsed potential.^[^ ^52^ ^]^ Specifically, when Cu_50_Ni_50_ alloy cathode is employed, the optimized pulse condition leads to in situ formation of Cu/Cu_2_O and Ni/Ni(OH)_2_ sites, which accelerate the rate‐determining step of NO_3_^−^‐to‐NO_2_^−^ and active hydrogen species adsorption (Figure 2c), respectively. As a result, an impressive NH_3_ YR of 583.6 µmol cm^−2^ h^−1^ and an FE of 88.0% were obtained, while under constant potential, the maximum NH_3_ YR was only 104.8 µmol cm^−2^ h^−1^, with an unsatisfied FE of 54.5%.

In another work, Zhou et al. used pulsed electrolysis to modulate the chemical state of a Cu/Cu_2_O cube catalyst by periodically applying oxidative potential pulses.^[^ ^53^ ^]^ Static condition yielded a NH_3_ FE of 93.9%. In contrast, pulsed electrolysis decreased the FE of NH_3_ to 70% and increased SE of NO_2_^−^ to 3.9%. Of note, the Cu_2_O of as‐prepared Cu/Cu_2_O underwent a structural transformation into oxide‐derived Cu^0^ (0D‐Cu) during applied potential cycling. These findings indicate that both product selectivity and active phase are potentially dependent: at lower potentials, the Cu/Cu_2_O interface preferentially facilitates the reduction of NO_3_^−^ to NO_2_^−^, while at higher potentials, 0D‐Cu serves as the active phase for deep hydrogenation of NO_2_^−^ for NH_3_ generation (Figure 2d).

In another representative study, a Co‐based electrocatalyst was observed undergoing reversible surface reconstruction via pulsed electrolysis, facilitating the in situ formation of active Co(III) species and Co_3_O_4_/CoOOH phases on working and counter electrodes, respectively.^[^ ^54^ ^]^ In this regard, the dynamic modulation enhanced NO_3_^−^RR activity while suppressing competing reactions. Compared to static electrolysis, the pulsed mode on Co‐based electrocatalyst in a one‐component cell achieved a significantly improved peak YR of 1500.9 µmol cm^−2^ h^−1^ with a comparable FE of 92.6%.

#### 3.1.2 Electrocatalyst Regeneration

In a prolonged electrochemical reaction, the electrocatalyst performance often declines due to the accumulation of poisoning intermediates or side products.^[^ ^55^, ^56^ ^]^ Periodic modulation of the applied potential can regenerate the catalyst surface by oxidizing and removing surface‐bound species, contributing to restoring active sites that may otherwise deactivate under steady‐state conditions.^[^ ^57^, ^58^ ^]^ A compelling example is the NiPr‐TPA‐COF catalyst, synthesized from 2D covalent organic frameworks (2D COF) and nickel porphyrin.^[^ ^59^ ^]^ Under static electrolysis, the accumulation of side products on the catalyst surface led to active site blockage and a subsequent decline in current density. To mitigate this, a pulsed potential strategy was employed, alternating between a reductive step at −1.38 V for 1000 s (enabling NO_3_^−^RR) and a brief oxidative step at +0.4 V for 1 s (oxidizing and removing accumulated side products). This approach enabled periodic surface regeneration without compromising current density or NH_3_ selectivity, as the current density consistently returned to its initial value following each anodic pulse. Consequently, an outstanding SE (90%), YR (2.5 mg h^−1^ cm^−2^), turnover frequency (up to 3.5 s^−1^), and stability (20 000 s) were collectively achieved.

#### 3.1.3 Electrocatalyst De‐Passivation

During electrochemical reactions, surface passivation of electrocatalysts can occur due to oxide formation and metal dissolution, leading to decreased activity. Pulsed electrolysis offers an effective strategy to mitigate passivation by periodically removing surface contaminants and soluble intermediates, thereby freeing up active sites of the catalyst surface.^[^ ^60^ ^]^ As a prime example, the role of the Co(II)/Co(III) redox cycle is investigated in NO_3_^−^RR under pulsed electrolysis.^[^ ^61^ ^]^ The alternating potential suppressed the redeposition of cobalt species (Co^δ+^) on the counter electrode, which otherwise leads to passivation and side reactions such as HER and NH_3_OR. The results show a high NH_3_ YR of 1.4 mmol cm^−2^ h^−1^ and FE of 91.7%. The results demonstrate the crucial role of the pulse‐driven Co(II)/Co(III) redox cycle for improved NO_3_^−^RR electrolysis and the dissolution and redeposition of cobalt species (Co^δ+^) for blocking anodic NH_3_OR (Figure 2e).

### 3.2 Reaction Microenvironment Optimization

#### 3.2.1 Regulation of Key Intermediates and Suppression of Side Reactions

The electrochemical interface constitutes a dynamic landscape, where the spatial and temporal distribution of ions, reaction intermediates, and local pH critically governs reaction pathways and product selectivity.^[^ ^62^, ^63^ ^]^ Pulsed electrolysis offers a powerful strategy to dynamically tune the interfacial environment, enhancing the availability and reactivity of key intermediates while suppressing undesired side reactions.^[^ ^64^, ^65^ ^]^

**Figure 3:**
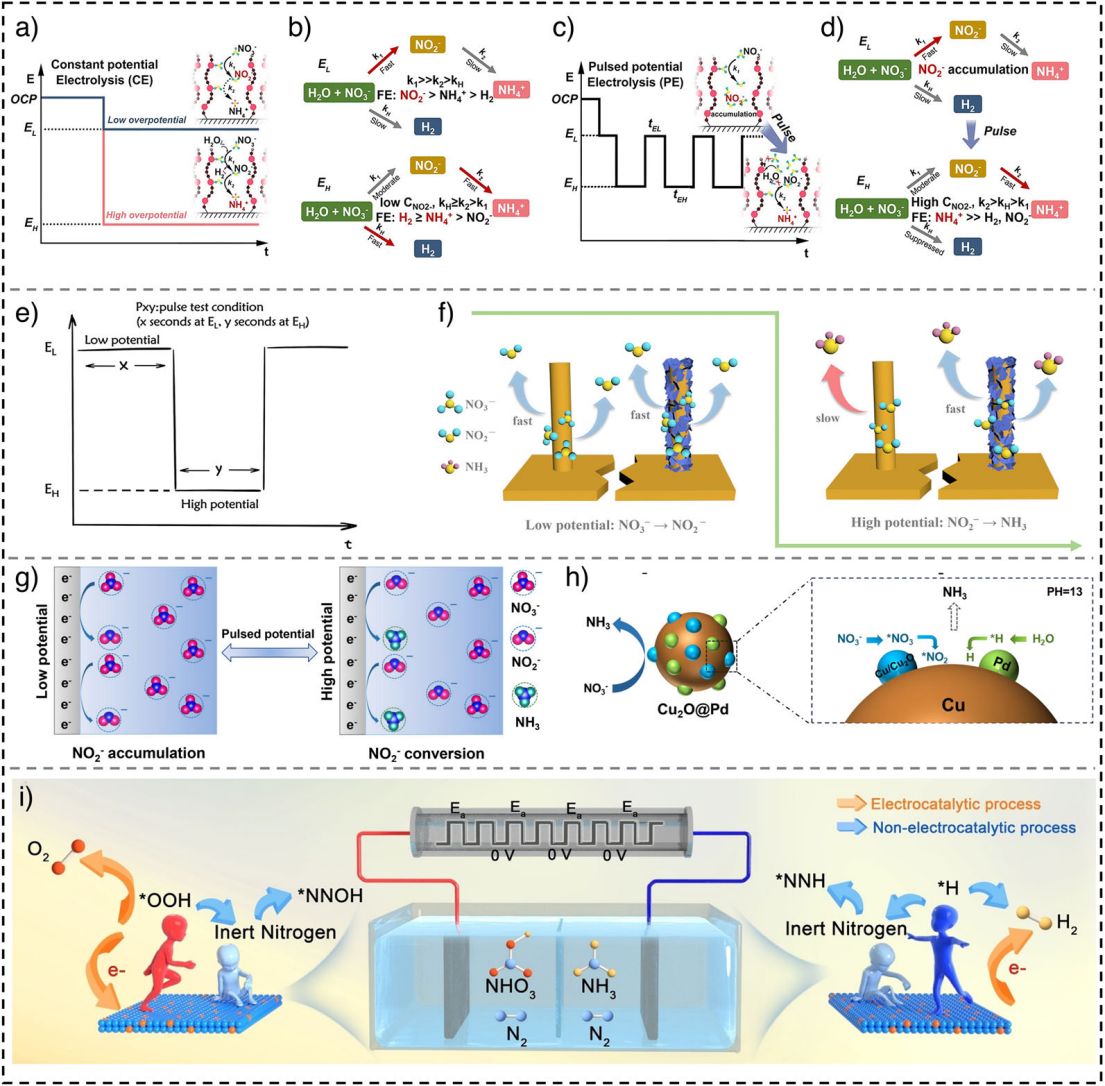
A schematic of a) and c) reaction pathways and b) and d) reaction mechanism under static and pulsed electrolysis (reproduced with permission from Ref. [66] Copyright © 2023 ACS), e) pulsed test schematic, f) proposed mechanism of NO_3_^−^RR on Co@Cu NW and Cu NW (reproduced with permission from Ref. [67] Copyright © 2024 ACS), g) pulsed potential influence on electrocatalyst environment, h) proposed tandem reaction on Cu_2_O@Pd at pH 13 (reproduced with permission from Ref. [39] Copyright © 2024 Elsevier), and i) diagram of nitrogen fixation process in NH_3_ and HNO_3_ production (reproduced with permission from Ref. [33] Copyright © 2023 Wiley‐VCH).

Li et al. designed a Cu single‐atom gel (Cu SAG) electrocatalyst, leveraging the NO_2_^−^ selectivity of isolated Cu sites and the aerogel's hierarchical porosity to enhance NO_3_^−^RR performance.^[^ ^66^ ^]^ Under potentiostatic electrolysis (Figure 3a,b), the reaction proceeds through two sequential steps: NO_3_^−^ to NO_2_^−^ (with the rate constant *k*_1_) and NO_2_^−^ to NH_4_⁺ (with the rate constant *k*_2_), with low overpotentials (*E*_L_) favoring *k*_1_ and high overpotentials (*E*_H_) enhancing *k*_2_. However, high *E*_H_ also promotes HER due to the excessive production of active H species via water dissociation. Pulsed electrolysis alternating between *E*_L_ and *E*_H_ facilitates NO_2_^−^ accumulation and conversion within the Cu SAG channels, while the locally enriched NO_2_^−^ suppresses HER by favoring its own reduction (Figure 3c,d). Optimized pulsed protocol yielded the highest FE of ∼97%, nearly doubling that of potentiostatic electrolysis, by effectively cascading NO_2_^−^ conversion while minimizing HER side reaction. In a related study based on Co@Cu nanowire catalyst,^[^ ^67^ ^]^ pulsed electrolysis was used as a strategy to temporally decouple NO_3_^−^RR steps across Cu and Co phases, optimizing selectivity and performance (Figure 3e,f). At low potential, the Cu phase accumulates NO_2_^−^, which is subsequently converted to NH_3_ at high potential by the Co phase, enabling time‐separated tandem catalysis. Remarkably, the peak NH_3_ YR and FE can be tuned via modulating high‐potential duration.

In another study, Dou et al. reported Cu_2_O@Pd as a tandem electrocatalyst for NO_3_^−^RR,^[^ ^39^ ^]^ achieving an FE of 81.2%, YR of 1.08 mg h^−1^ cm^−2^, and SE of 81.5% under optimized pulsed conditions. The enhanced performance arises from the synergistic roles of Cu in NO_3_^−^ activation and Pd in hydrogen adsorption (Figure 3g,h). Note that the in situ Fourier transform infrared spectroscopy (FTIR) studies under pulsed operation revealed increased intensities of N‐containing intermediates, indicating improved stabilization and stepwise conversion compared to potentiostatic electrolysis. The data support a tandem mechanism that involves deoxygenation steps facilitated by Cu (100) facet on the in situ generated Cu/Cu_2_O, and deep hydrogenation steps facilitated by Pd for active hydrogen generation via water dissociation. In a separate work, Yang et al. engineered a (Co_0.83_Ni_0.16_)_2_Fe layered double oxide (LDO) electrocatalyst by Ni^2+^ substitution in Co/Fe LDOs, achieving a high NH_3_ YR of 50.4 mg h^−1^ cm^−2^ and FE of 97.8% under pulsed electrolysis.^[^ ^68^ ^]^ In situ Raman spectroscopy and computational analyses showed that Ni incorporation modulates the electronic structure of Co, enhancing *H adsorption and accelerating water dissociation, thereby promoting NO_2_^−^ hydrogenation. Pulsed electrolysis further improved NO_3_^−^ distribution near the catalyst surface and synchronized the kinetics of NO_3_^−^‐to‐NO_2_^−^ and NO_2_^−^‐to‐NH_3_ steps, contributing to the superior performance.

Additionally, a recent study demonstrated that pulsed electrolysis markedly enhances overall nitrogen fixation, enabling the co‐production of HNO_3_ and NH_3_.^[^ ^33^ ^]^ Atomically dispersed Fe on TiO_2_ (Fe‐TiO_2_) showed superior performance under pulsed conditions, achieving a HNO_3_ yield of 7055.81 µmol h^−1^ g^−1^_cat_ and a 44.94‐fold increase in FE compared to static electrolysis. Similarly, NH_3_ production reached 12 868.33 µmol h^−1^ g^−1^_cat_, accompanied by a 7.8‐fold improvement in FE. These enhancements are attributed to the suppression of competing HER and OER, improved N_2_ accessibility, and the activation of a nonelectrocatalytic pathway during low‐voltage pulses, wherein ^*^OOH intermediates react with ^*^N_2_ without electron transfer (Figure 3i).

#### 3.2.2 Enhanced Mass Transfer

Pulsed electrolysis improves mass transfer for NO_3_^−^RR via periodic current interruption, mitigating concentration polarization by facilitating diffusion layer replenishment.^[^ ^69^ ^]^ Alternating off‐phases (enhancing NO_3_^−^ ion diffusion) and on‐phases (enabling high current densities without polarization) optimize reactant availability at active sites, elevating reaction efficiency and selectivity.^[^ ^39^ ^]^ This approach suppresses parasitic reactions, such as HER, while performance hinges on tailored pulse parameters (frequency, duty cycle) to harmonize diffusion kinetics and electron transfer, necessitating system‐specific optimization.^[^ ^38^ ^]^

**Figure 4:**
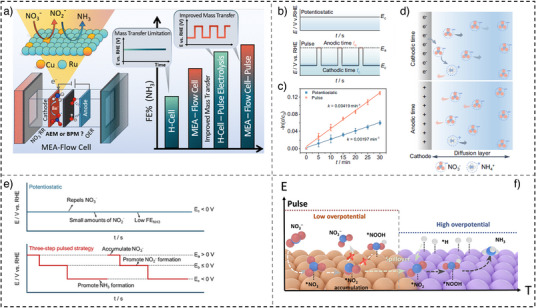
a) A schematic illustration of set‐up of MEA‐flow cell and comparison of static and pulsed nitrate electrolysis (reproduced with permission from Ref. [71] Copyright © 2024 ACS), b) static and pulsed set‐up, c) the linearized pseudo‐first‐order kinetic profiles under static and pulsed conditions, d) an illustration of pulsed electrolysis influence on the mass transport (reproduced from Ref. [34] published in 2023, under the terms of the Creative Commons Attribution license (CC BY 4.0)), e) a schematic of the ionic distribution along with the steps (reproduced with permission from Ref. [72] published in 2025, under the terms of the CC BY 4.0), and f) An illustration of ammonia electrosynthesis under pulsed potential (reproduced with permission from Ref. [73] Copyright © 2025 Wiley‐VCH).

In one work, planar polycrystalline Ti foil is used in flow cell reactors with varied volumetric flow rates (5–115 mL min^−1^) to study the influence of mass transport and interfacial environment.^[^ ^70^ ^]^ Electrochemical evaluation, continuum modeling, and in situ FT‐IR spectroscopy revealed that enhanced mass transport improves NO_3_^−^RR activity, while interfacial pH governs product selectivity. Pulsed electrolysis was found to lower interfacial pH by modulating hydroxide accumulation during cathodic steps, thereby increasing proton availability and favoring NH_3_ formation. Compared to potentiostatic electrolysis, pulsed operation shifted the dominant product from NO_3_^−^ to NH_3_, achieving a 3.4‐fold increase in SE. Additionally, Cu*_x_* Ru*_y_* alloy catalysts supported on carbon black were evaluated for NO_3_^−^RR in both H‐cell and membrane electrode‐assembly configurations employing an anion exchange membrane (MEA‐AEM) configuration (Figure 4a).^[^ ^71^ ^]^ Pulsed electrolysis significantly enhanced NO_3_^−^RR performance in both H‐cell (FE of 92.9% and YR of 0.15 mmol h^−1^ cm^−2^) and MEA‐AEM setup (FE of 94.94% and YR of 0.141 mmol h^−1^ cm^−2^) in comparison with the potentiostatic electrolysis conditions. Notably, the MEA‐AEM cells generally exhibit lower cell voltages than H‐cells, which is attributed to the enhanced NO_3_^−^ ion transport at the catalyst/electrolyte interface. The enhanced NO_3_^−^‐to‐NH_3_ conversion arises from pulsed electrolysis promoting tandem catalysis via accelerated NO_2_^−^‐to‐NH_3_ conversion, dynamic phase transitions of Ru‐to‐RuO_*x*_ during the anodic step, microenvironment modulation, and optimized reaction intermediates adsorption.

In another study, carbon‐supported RuIn_3_ intermetallic compounds (RuIn_3_/C)^[^ ^34^ ^]^ demonstrated excellent NO_3_^−^RR performance under optimized pulsed electrolysis, achieving an FE of 97.6% and a YR of 2.7 mmol h^−1^ mg_Ru_^−1^, double that of potentiostatic conditions (Figure 4b). The incorporation of In modulates the electronic structure of Ru, while pulsed potential enhances interfacial ion diffusion and reaction kinetics (Figure 4c). Specifically, pulsed electrolysis improves mass transport by replenishing NO_3_^−^ in the diffusion layer and facilitating NH_3_ product desorption, while also increasing local NO_3_^−^ concentration and optimizing *NO intermediate adsorption (Figure 4d). Overall, pulsed operation significantly enhances kinetics, mass transfer, intermediate stabilization, and product selectivity, while effectively suppressing side reactions (e.g., HER).

Moreover, a recently proposed three‐step pulsed strategy markedly enhances the electrochemical reduction of NO_3_^−^ to NH_3_ (Figure 4e).^[^ ^72^ ^]^ The process proceeds through 1) application of a positive potential (0.3 V versus RHE) to enrich negatively charged NO_3_^−^ ions at the interface; 2) a mild negative bias (0.0 V versus RHE) that selectively reduces the accumulated NO_3_^−^ to NO_2_^−^; and 3) strong negative pulses (−0.6 to −0.7 V versus RHE) that drive the rapid hydrogenation of NO_2_^−^ intermediates to NH_3_. The localized accumulation‐depletion behavior of reactants and intermediates is corroborated by theoretical simulations and operando characterizations. In another example, Li et al. demonstrated that pulsed electrolysis can promote a spillover mechanism on a Janus Cu@Co/NC dual‐site electrocatalyst (Figure 4f).^[^ ^73^ ^]^ During low‐potential phases, pulsed operation enhances NO_3_^−^ distribution, thereby improving mass transfer and facilitating *NO_2_ accumulation on Cu sites. Subsequent potential switching, coupled with the Janus interface, enables *NO_2_ spillover from Cu to Co sites, optimizes *H supply, and accelerates intermediate hydrogenation, ultimately maximizing NH_3_ production. Specifically, a summary of NO_3_−to‐NH_3_ via pulsed electrolysis for this section is provided in Table 1.

**Table 1:** Summary of NO_3_^−^‐to‐NH_3_ via pulsed electrolysis. FE and SE are reported in % and YR is given in µmol cm^−2^ h^−1^.

		Pulse profile						
	Electrocatalyst	E_ca_ (V vs. RHE) / t_ca_ (s)	E_an_ (V vs. RHE) / t_an_ (s)	Static potential (V vs. RHE)	Performance under pulsed electrolysis	Performance under static potential	NO_3_^−^ concentration (M)/supporting electrolyte	References
Electrocatalyst modulation								
New active phase formation	CuO formed from CuO/Cu_2_O	−1.2 to 1.6/2	1.0/2	Unspecified	SE = 95 FE = 100	SE = 72	0.0015–0.0085/0.05 M Na_2_SO_4_	[51]
	Cu/Cu_2_O and Ni/Ni(OH)_2_ formed from Cu and Cu‐Ni alloys	−1/5	1.4/5	−0.5	FE = 88.0 YR = 583.6	FE = 54.5 YR = 104.8	0.1/0.01 M KOH + 0.5 M Na_2_SO_4_	[52]
	0D‐Cu formed from Cu/Cu_2_O cubes	−0.8/180	0.7/180	−0.9	FE = 80	FE = 93.9 YR = 219.8	0.1/0.1 M PBS	[53]
	Co_3_O_4_/CoOOH from Co‐Ni	−0.9/5	1.2/5	−0.9	FE = 92.6 YR = 1500.9	FE = 94 YR = 679.0	0.1/1 M KOH	[54]
Electrocatalyst regeneration	NiPr‐TPA‐COF	−1.38/1000	0.4/1	−1.46	FE ∼ 73%	YR = 147 SE = 90	0.1/0.5 M K_2_SO_4_	[59]
Electrocatalyst de‐passivation	Co‐based catalysts	−0.5/5	0.4–1.4/5	−0.5	FE = 91.7 YR = 1400	FE = 52.5 YR = 179.3	0.1/1 M KOH	[61]
Reaction microenvironment optimization								
Regulation of key intermediates and suppression of side reactions	Cu SAG	−0.5/1.0	−0.8/1.0 –5.0	−0.8	FE = 97 YR = 30.84	FE = 78 YR = 25.84	0.02/0.1 M PBS	[66]
	Co@ Cu nanowire	−0.2/1	−0.7/1–5	−0.4	FE = 88.6 YR = 302.37	FE = 90	0.1/0.5 M Na_2_SO_4_	[67]
	Cu_2_O@Pd	0.12/5	−0.48/8	−0.53	FE = 81.2 YR = 63.42 SE = 81.5	FE = 37.4 YR = 36.40 SE = 48.7	0.01/0.1 M NaOH	[39]
	(Co_0.83_Ni_0.16_)_2_Fe LDOs	−0.42/20	1.24/0.2	−0.42	FE = 97.8 YR = 2959.31	FE = 90 YR = 2436.73	0.1/1 M KOH	[68]
	Fe─TiO_2_	0 V vs. SHE/0.25	3.5 V vs. SHE/0.25	3.5	FE _NH3 _= 8.85 YR _NH3 _= 12 868.33 µmol h^−1^ g^−1^	FE _NH3 _= 1.13 YR _NH3 _= 7185.00 µmol h^−1^ g^−1^	N_2_‐saturated/3 M H_2_SO_4_	[33]
					FE _NO3_− = 8.09% YR _NO3_− = 7055.81 µmol h^−1^ g^−1^	FE _NO3_− = 0.18% YR _NO3_− = 673.33 µmol h^−1^ g^−1^		
Enhanced mass transfer	Ti foil	−1.0/10	0.6/10	−1.0	SE_NH3_/SE_NO2_− = 1.7	SE_NH3_/SE_NO2_− = 0.5	0.01/0.01 M NaClO_4_	[70]
	Cu*_x_* Ru*_y_*	−0.2/9.5	0.6/1	−0.4	FE = 94.94 YR = 141	YR = 40	0.01–0.5/0.1 M KOH	[71]
	RuIn_3_/C	−0.1/4	0.6/0.5	0	FE = 97.6 YR = 2.7 mmol h^−1^ mg_Ru_^−1^	FE = 65.8 YR = 1.1 mmol h^−1^ mg_Ru_^−1^	0.01/0.1 M KOH	[34]
	CuNW/Cu foam	0.3/1	−0.6 to −0.7/20	−0.7	FE = 90	FE = 25.09	10 mM/1.0 M KOH	[72]
	Janus Cu@Co/NC (J‐Cu@Co/NC	0.1/2	−0.3/0.5–3	−0.4	FE = 98.32 YR = 748.39	FE = 73.62 YR = 500.88	0.01/0.1 M KOH	[73]

## 4 Coupling Reductive Nitrogen Species Activation with Carbon Species Reduction for C─N Bond Formation

Pulsed electrolysis for C─N coupling by reductive nitrogen species activation with carbon species reduction remains a nascent area of research, with only a limited number of studies reported to date. By modulating the applied potential, this technique enables dynamic control over the reaction environment, primarily through the regulation of intermediate species.^[^ ^29^ ^]^ Given its early stage of development in this context, potential advantages over electrocatalyst modulation have yet to be thoroughly investigated. These unexplored aspects represent promising avenues for future research as the field continues to expand. This section provides a seminal overview of how pulsed electrolysis affects C─N bond formation by dynamically modulating the reaction environment (Table 2).

**Table 2:** Summary of C─N coupling via pulsed electrolysis. FE and SE are reported in %. In Ref. [36], YR is reported in %.

	Pulse profile							
Electrocatalyst	E_ca_ (V vs. RHE) / t_ca_ (s)	_an_ (V vs. RHE) / t_an_ (s)	Static potential (V vs. RHE)	Product	Performance under pulsed electrolysis	Performance under static potential	Electrolyte	References
Electrosynthesis of amide								
FeTPP/CNTs	−0.8/2	−0.2/2	−0.8	Urea	YR = 53.86 µg mg^−1^ C^−1^ FE = 27.70	FE = 19.16	0.2 M KHCO_3_ + 0.1 M KNO_3_ saturated with CO_2_	[74]
Polycrystalline gold electrode	−0.4/1	0. 2/0.2	−0.3	Urea	FE = 60.4	FE = 30.8	0.1 M KNO_3_ + 0.1 M KHCO_3_ saturated CO_2_	[75]
CuSiO_*x*_	0/20	OCP/10	−0.6	Urea	FE = 80 SE = 79.01% YR = 1606.1 µg h^−1^ mg_cat._^−1^	FE = 57 YR = 656.4 µg h^−1^ mg_cat._^−1^	0.1 M KNO_3_ + KHCO_3_ saturated with CO_2_	[76]
Electrosynthesis of amine								
Low‐coordinated Cu nanocoral (LC‐Cu NC)	−0.7 V vs. Hg/HgO/1	0.6 V vs. Hg/HgO/2	−1.1 V vs. Hg/HgO	Aniline	YR = 72%	YR = 50%	0.25 M PBS + MeOH (2:1 v/v) 2.0 mmol NaNO_2_ 0.1 mmol phenylboronic acid	[36]

### 4.1 Electrosynthesis of Amide (with a Focus on Urea)

**Figure 5:**
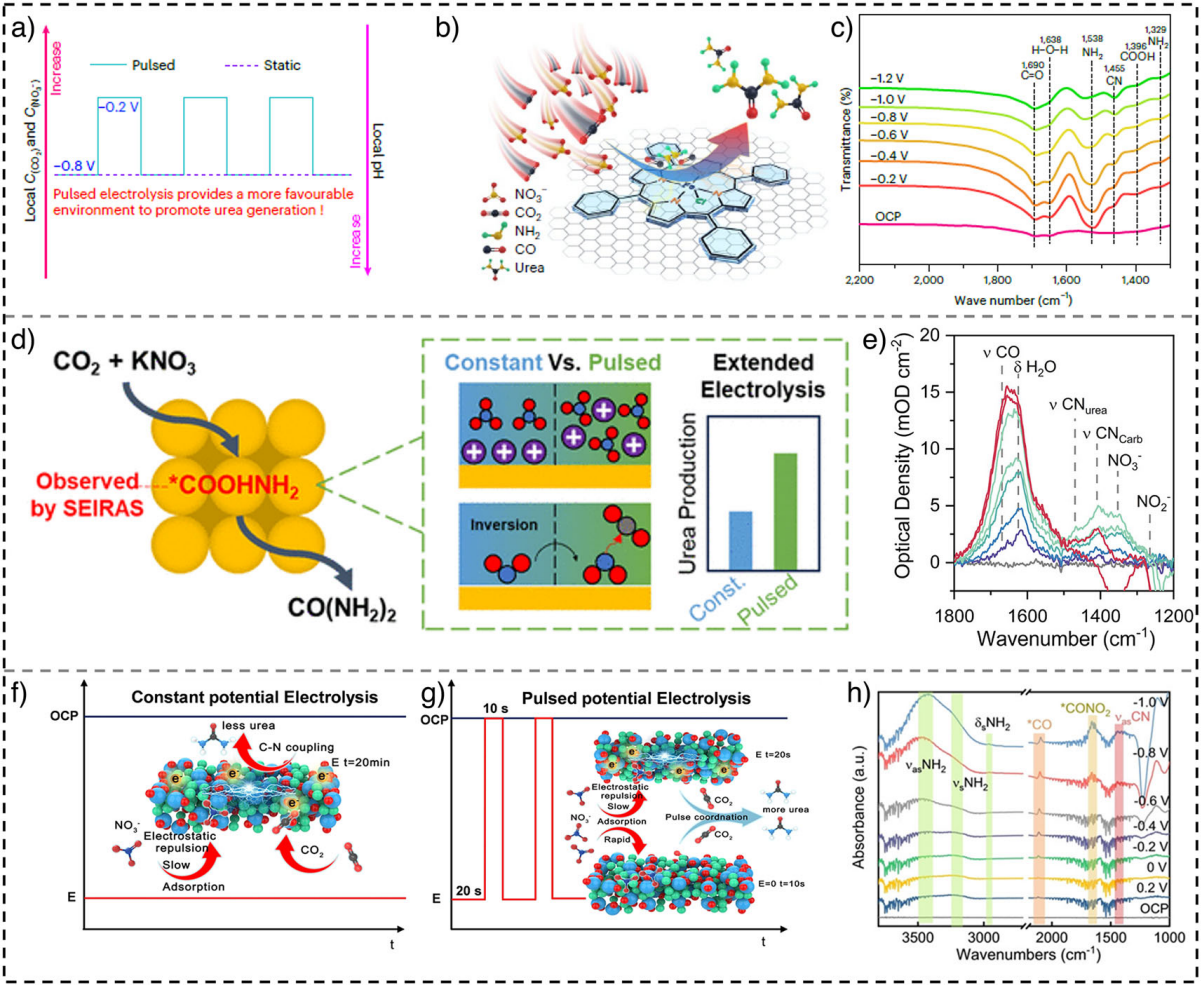
a) A schematic of influence pulsed set‐up on reaction environment, b) schematic representation of the C─N coupling over Fe‐TPP/CNTs under the pulsed electrolysis, c) operando ATR‐SEIRAS spectroscopy measurements for the C─N coupling (reproduced with permission from Ref. [74] Copyright © 2024 Springer Nature), d) a comparison of C─N coupling under static and pulsed conditions, e) in situ SEIRAS at different potential (reproduced with permission from Ref. [75] Copyright © 2023 ACS), a schematic of C─N co‐reduction under f) static and g) pulsed potential, and h) in situ SEIRAS on CuSiO*_x_* at different voltages (reproduced with permission from Ref. [76] Copyright © 2024 Wiley‐VCH).

In a representative study, Fe‐tetraphenylporphyrin (Fe‐TPP) was utilized as a molecular catalyst for urea electrosynthesis via C─N coupling from NO_3_^−^RR and CO_2_ reduction reaction (CO_2_RR).^[^ ^74^ ^]^ Pulsed potential application enhanced the SE toward key intermediates (*CO from CO_2_RR and *NH_2_ from NO_3_^−^RR, Figure 5a,b), while suppressing side reactions (e.g., HER). It also lowered the activation energy for C─N bond formation, improving kinetics and urea yield. Under optimized conditions, a maximum FE of 27.70% and a urea YR of 53.86 µg mg^−1^ C^−1^ were achieved. In contrast, constant potential at −0.8 V versus RHE yielded a lower FE of 19.15%. The attenuated total reflectance surface‐enhanced infrared absorption spectroscopy (ATR‐SEIRAS) analysis across 0 to −1.2 V versus RHE (Figure 5c) showed a peak at 1455 cm^−1^ (C─N bond) peaking at −0.8 V, consistent with peak FE, while the *NH_2_‐associated peak at 1538 cm^−1^ decreased at more negative potentials, indicating its consumption during C─N coupling.

In another related study, a polycrystalline gold electrode was employed as an electrocatalyst for urea electrosynthesis via tandem electrolysis of NO_3_^−^RR and CO_2_RR.^[^ ^75^ ^]^ The results showed that pulsed electrolysis disrupts the electrochemical double layer, thereby enhancing mass transport of reactants (e.g., CO_2_ and NO_3_^−^) to the catalyst surface. In particular, NO_3_^−^ ions were found to migrate more efficiently toward the electrode, modifying the local reaction environment (Figure 5d). Under pulsed conditions, a high FE of 60.4% was achieved; while a maximum urea FE of 30.8% was achieved with potassium (K^+^)‐based electrolytes at −0.3 V versus RHE. In addition, time‐resolved SEIRAS confirmed that pulsed potentials improve NO_3_^−^ transport due to electrostatic attraction and repulsion effects between the polarized electrode and electrolyte species (Figure 5e). Furthermore, DFT calculations indicated that potential modulation facilitates the desorption of adsorbed species, particularly NO_3_^−^ and NO_2_^−^ anions, through increased electrostatic repulsion, thereby exposing active sites for C─N coupling on the electrode surface.

Furthermore, Qiu et al. developed a CuSiO_*x*_ nanotube catalyst featuring abundant atomic Cu^0^─O─Si interfacial sites for the electrosynthesis of urea from NO_3_^−^RR and CO_2_RR under pulsed electrolysis conditions.^[^ ^76^ ^]^ Electrostatic interactions typically hinder anion reduction at the cathode (Figure 5f); however, this challenge can be effectively addressed by periodically alternating the applied potential (Figure 5g). The study reported a urea YR of approximately 1606.1 µg h^−1^ mg_cat_^−1^, a SE of 79.01%, and an FE retention of 80% over 80 h of operation. Under potentiostatic conditions, an FE of 57% was achieved at −0.2 V versus RHE, while the highest urea YR of 656.4 µg h^−1^ mg_cat_^−1^ was obtained at −0.6 V versus RHE. Theoretical calculations and in situ ATR‐SEIRAS analyses revealed that the rate‐determining step in urea formation involves C─N coupling between hydrogenated *NO_2_ intermediate from NO_3_^−^RR and *CO intermediate from CO_2_RR on the CuSiO_*x*_ surface (Figure 5h). Pulsed electrolysis was shown to alleviate mass transport limitations by increasing the local concentration of *NO_2_^−^ near the electrode. The superior performance is attributed to the dynamic modulation of the electrochemical environment under pulsed conditions, which enhances NO_3_^−^ ion migration and facilitates the key C─N coupling step.

### 4.2 Electrosynthesis of Amine

**Figure 6:**
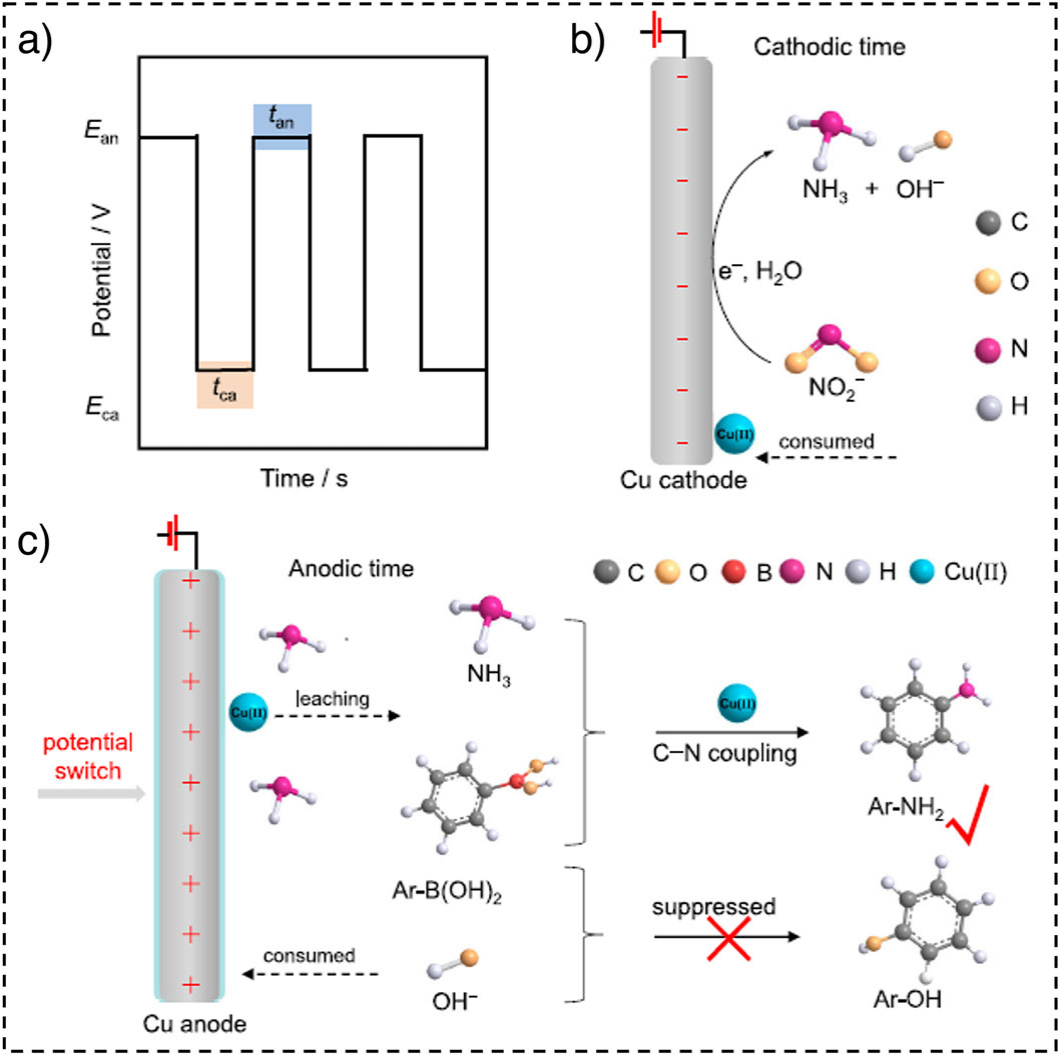
a) Pulsed potential profile, b) NO_2_^−^ reduction prior to pulsing, c) Cu(II) formation and Chan–Lam coupling at anodic potential (*E*_an_) (reproduced from Ref. [36] published in 2023, under the terms of the CC BY 4.0).

He et al. proposed the use of a low‐coordinated copper nanocoral (LC‐Cu NC) cathode for the electrochemical NO_2_^−^‐to‐NH_3_, followed by C─N coupling by arylboronic acids to synthesize arylamines.^[^ ^36^ ^]^ A maximum arylamine YR of approximately 72% was achieved at the optimized pulsed condition (Figure 6a), where Cu is oxidized in situ to Cu(II), a well‐known active species in the Chan–Lam coupling reaction.^[^ ^77^, ^78^ ^]^ In situ electrochemical ATR‐FTIR spectroscopy identified *NO_2_, *NO, and *NH_2_ as key intermediates involved in the electrosynthesis of arylamines. Moreover, electron paramagnetic resonance (EPR) spectroscopy confirmed the formation of a catalytic Cu(II)─NH_3_ complex, which is critical for facilitating the Chan–Lam coupling mechanism. These findings underscore the multifunctional role of pulsed electrolysis (Figure 6b,c), which includes: 1) in situ generation of the Cu(II) catalyst, (2) suppression of phenol byproduct formation, and (3) modulation of substrate and intermediate concentrations within the EDL through rapid polarity switching. Additionally, the reversible transition between Cu(II) release during the anodic pulse and redeposition during the cathodic pulse further highlights the dynamic nature of this approach.

## 5 Outlook and Emerging Directions

### 5.1 Established Pulsed Electrolysis Systems

**Machine learning (ML)‐guided catalyst manipulation and pulse parameter optimization**: Pulsed electrolysis imposes unique structural and performance demands on catalysts, requiring them to tolerate rapid cycling without degradation or loss of active sites, while maintaining activity and selectivity.^[^ ^23^, ^79^ ^]^ Beyond catalyst composition, surface structure and morphology critically influence intermediate adsorption and transformation, underscoring the need for materials with robust stability and adaptable surface chemistry.^[^ ^29^ ^]^ Additionally, pulse parameters are intricately coupled with catalyst properties, necessitating tailored designs that can accommodate nonequilibrium states and transient intermediates. Moving beyond conventional trial‐and‐error methodologies, ML‐guided approaches offer powerful tools not only for performance optimization but also for enhancing catalyst stability. For instance, ML models can elucidate correlations between pulse profiles and degradation pathways, thereby enabling the prediction and design of stress‐tolerant catalyst architectures. Such ML‐guided catalyst manipulation significantly reduces experimental time and effort, with representative examples including NORR‐to‐NH_3_ on CuNi alloy^[^ ^80^ ^]^ and Ag‐based single‐atom alloys,^[^ ^81^ ^]^ NRR on BaH_2_,^[^ ^82^ ^]^ and MXene‐based single atom,^[^ ^83^ ^]^ NO_3_^−^RR on CuPd nanocubes^[^ ^84^ ^]^ and CuCo alloy,^[^ ^85^ ^]^ as well as urea synthesis accelerated by theoretical simulation.^[^ ^86^ ^]^ Moving forward, it is essential to conduct systematic investigations into the compatibility of specific catalyst manipulation with tailored pulse modulation strategies. In this context, incorporating design of experiments (DoE) methodologies into the pulse modulation framework offers particular advantages. When coupled with ML, DoE can further accelerate and refine the optimization of both catalyst design and pulse engineering, enabling comprehensive and efficient exploration of the multidimensional parameter space.

**Role of potential polarity and electrolyte**: Recent advances in pulsed electrolysis have highlighted that the performance and selectivity are highly sensitive to the surrounding electrochemical environment and the parameters involved in waveform design. To this end, switching between unipolar and bipolar pulse modes (e.g., both positive/positive or negative/negative potential) enables dynamic control over the electrochemical interface.^[^ ^33^, ^51^, ^74^ ^]^ It fine‐tunes the local reaction microenvironment, suppresses competing side reactions, and improves product selectivity. Simultaneously, the rational regulation of the electrolyte microenvironment, in particular, the influence of cations^[^ ^87^ ^]^ has attracted increasing attention for its ability to enhance the NRR by facilitating N≡N bond cleavage^[^ ^88^ ^]^ and the NO_3_^−^RR by electrostatically stabilizing NO_3_^−^ adsorption and accelerating water dissociation for hydrogenation.^[^ ^89^, ^90^, ^91^ ^]^ Together, these insights underscore the importance of rationally engineering both the potential waveform and electrolyte environment to unlock new selectivity mechanisms and optimize electrocatalytic performance under pulsed conditions.

**In situ methods for mechanistic decoding**: Understanding the mechanistic intricacies of reductive nitrogen species activation is essential for the rational design of next‐generation electrocatalytic systems. In this regard, the deployment of in situ spectroscopic and microscopic electrochemical techniques under pulsed electrolysis conditions offers a powerful means to capture real‐time reaction dynamics. For example, FTIR^[^ ^92^, ^93^ ^]^ and Raman spectroscopy^[^ ^93^, ^94^ ^]^ provide structural insights into surface‐formed species and adsorbates, complemented by differential electrochemical mass spectrometry (DEMS).^[^ ^95^, ^96^ ^]^ X‐ray photoelectron spectroscopy (XPS)^[^ ^97^ ^]^ reveals surface elemental composition and oxidation‐state changes under operating conditions, while X‐ray absorption spectroscopy (XAS)^[^ ^40^, ^98^ ^]^ elucidates local chemical transformations of the probed element, including electronic structure, bond lengths, oxidation states, and coordination environments. Further approaches include scanning electrochemical microscopy (SECM),^[^ ^99^ ^]^ which sheds light on surface adsorbates, and transmission electron microscopy (TEM),^[^ ^100^ ^]^ which tracks dynamic structural and morphological evolution at the nano‐to‐atomic scale. Additionally, the use of in situ isotope‐labeling strategies, employing isotopically enriched species, such as ^13^CO_2_, ^15^NO_3_^−^, ^15^NO_2_^−^, ^15^N_2_O, ^15^NO, and ^15^N_2_, combined with carefully designed control experiments, will enable detailed tracking of the generation, transformation, and interaction of reactive intermediates under pulsed conditions.

### 5.2 Emerging Themes for Pulsed Electrolysis

**Integration with biomass oxidative processes**: Replacing the typically sluggish anodic oxygen evolution reaction (OER)^[^ ^101^ ^]^ with thermodynamically favorable biomass electro‐oxidation reactions presents a compelling opportunity to reduce overall energy consumption while simultaneously generating value‐added products at the anode.^[^ ^102^ ^]^ Recent examples have been reported for NRR coupled with 5‐hydroxymethylfurfural (HMF) oxidation,^[^ ^103^, ^104^ ^]^ NO_3_^−^RR‐coupled with oxidation of ethylene glycol,^[^ ^105^ ^]^ glycerol,^[^ ^106^, ^107^, ^108^, ^109^ ^]^ formaldehyde,^[^ ^110^, ^111^ ^]^ methanol,^[^ ^112^ ^]^ and glucose.^[^ ^113^ ^]^ In this context, the application of pulsed electrolysis in such coupled systems holds great promise for enhancing the synchronization between the cathodic and anodic half‐reactions, thereby further improving overall performance, particularly with respect to product selectivity. This hypothesis is further supported by a recent work on overall nitrogen fixation, where pulsed electrolysis enabled efficient coupling of NRR for NH_3_ with NOR for HNO_3_ production.^[^ ^33^ ^]^

**Electrosynthesis of hydroxylamine as a product or key intermediate**: As previously discussed, hydroxylamine is an important industrial feedstock. However, its electrosynthesis remains challenging due to the propensity of nitrogen species to undergo over‐reduction to NH_3_.^[^ ^8^ ^]^ Most pioneering efforts have focused on the electrochemical reduction of NO using single‐metal‐site catalysts,^[^ ^114^, ^115^, ^116^ ^]^ leaving significant potential for the application of pulsed electrolysis across a broader range of industrially relevant electrocatalysts and alternative nitrogen‐containing pollutants (e.g., NO_3_^−[^ ^117^ ^]^/NO_2_^−[^ ^118^ ^]^). Moreover, by regulating hydroxylamine as a key intermediate derived from nitrogen‐containing species, pulsed electrolysis is expected to facilitate cascade pathways for electrochemical C─N coupling. For example, this could enable the selective production of amino acids via co‐reduction with keto acids,^[^ ^119^, ^120^, ^121^, ^122^, ^123^ ^]^ and oximes via co‐reduction with ketones or aldehydes,^[^ ^124^, ^125^, ^126^, ^127^, ^128^ ^]^ thus expanding the synthetic utility of hydroxylamine under controlled electrochemical conditions. Notably, in organonitrogen electrosynthesis from CO_2_ and nitrogenous sources, the electrocatalytic C─N coupling reactions strongly rely on the gas–liquid–solid triple‐phase interface and interfacial water structure,^[^ ^129^ ^]^ suggesting that microenvironmental control under pulsed conditions can critically govern activity and selectivity.

**Photo‐(electro)chemical reductive nitrogen species conversion**: Although numerous achievements with satisfying YR and FE for NH_3_ production have been reported, the electrocatalytic NO_3_^−^RR process still necessitates high applied voltages in most cases, resulting in high electric energy consumption, which can be alleviated by the development of photoelectrodes.^[^ ^130^ ^]^ Notably, semiconductor photoelectrodes generate a built‐in potential under solar illumination, which can be leveraged to shift the operating potential to a more positive value. This principle has been successfully demonstrated in photoelectrodes for both HER^[^ ^130^ ^]^ and CO_2_RR.^[^ ^131^ ^]^ Nevertheless, the application of the photoelectrochemical (PEC) method for converting nitrogen species into NH_3_ and related products remains in its nascent stages. In a related work, Zhang et al.^[^ ^132^ ^]^ have pioneered the PEC NO_3_^−^‐to‐NH_3_ conversion using a Au‐decorated ordered silicon nanowire array photocathode, obtaining 95.6% of NH_3_ FE at 0.2 V versus RHE, which represents a more positive potential than the thermodynamic reduction potential of NO_3_^−^ by utilizing photovoltage. They proposed that the high FE could be attributed to the fact that both Si and Au surfaces are inactive for competing HER.

Furthermore, by integrating CO_2_RR with NO_3_^−^RR, semiconductor photoelectrodes have been demonstrated to efficiently enable PEC urea synthesis under solar light illumination. In a related work, Xu et. al.^[^ ^133^ ^]^ reported an efficient PEC method for urea synthesis by co‐reduction of CO_2_ and NO_3_^−^ over a Cu_2_O photocathode under AM 1.5 G irradiation, delivering urea formation rate of around 30 µmol g^−1^ h^−1^ and FE of 13% at a low external potential of −0.017 V versus RHE. Experimental data combined with theoretical calculations suggested that the adsorbed CO* and NO_2_* species are the key intermediates, and associated C─N coupling is the rate‐determining step.

### 5.3 Sustainability and Scalability

**Translating from lab to industrial scale**: Despite encouraging advances in pulsed electrochemical reduction of nitrogen species, translation to large‐scale application requires a deeper mechanistic understanding.^[^ ^134^ ^]^ Time‐resolved in situ characterization aforementioned, in combination with machine learning frameworks, will be critical to establish predictive design principles for pulse protocols. From an application perspective, current demonstrations achieve performance and operational lifetimes that remain far below industrial benchmarks (i.e., current densities > 500 mA cm^−2^ and operational stability > 1000 h). Bridging this gap will require systematic evaluation of catalyst stability, reproducibility, and selectivity under practical conditions, alongside standardized protocols and careful optimization of cell design and pulse parameters to minimize unintended side reactions and facilitate broader adoption.^[^ ^73^, ^111^ ^]^ Furthermore, although pulsed electrolysis allows temporal separation of adsorption, activation, and desorption steps, the challenges of maintaining consistent performance under fluctuating potentials should be carefully considered. In this regard, a comprehensive comparison of static versus pulsed electrolysis is provided in Table 3.

**Table 3:** Comparison of static and pulsed electrolysis for nitrogen species reduction.

Aspect	Static electrolysis	References	Pulsed electrolysis	References
Reaction pathway control	Limited; governed by steady‐state kinetics	[138]	Enhanced; dynamic modulation enables selective pathways	[66]
Intermediate stabilization	Often leads to accumulation or poisoning	[139]	Temporal separation allows better control of intermediates	[140]
Selectivity	Moderate; prone to side reactions	[141]	Improved; pulse timing can suppress undesired reactions	[33, 74]
Catalyst stability/reproducibility	Limited by deactivation during extended redox cycling	[142, 143]	Potentially improved via active site regeneration and new‐phase formation	[53]
Compatibility with renewable energy	Requires buffering/storage systems	[144]	Advantageously compatible with intermittent energy sources	[145]
Mechanistic understanding	Partly established	[146]	Progressively evolving by advanced in situ diagnostics and reactor design	[23]

**Techno‐economic analysis (TEA)**: Despite advancements in selectivity and energy efficiency, pulsed systems remain largely unevaluated in terms of TEA viability. Early‐stage assessments of steady‐state NH_3_ synthesis (NRR‐to‐NH_3_,^[^ ^135^ ^]^ NO_3_^−^RR‐to‐NH_3_,^[^ ^42^, ^136^ ^]^ NO_3_^−^RR‐to‐NH_3_ coupled with glycerol‐to‐formic acid^[^ ^108^ ^]^) or C─N coupling^[^ ^137^ ^]^ suggest that high energy demands, catalyst degradation, and process complexity are major cost drivers. Pulsed operation introduces further challenges in power delivery and system control, which may raise capital and operational expenditures unless addressed by integrated and modular designs. In this context, standardized metrics and dynamic TEA frameworks that account for variable input and pulse parameters are urgently needed.

**Hybrid solar/wind‐driven pulsed reactors**: Integrating pulsed electrolysis with intermittent renewable energy sources (e.g., solar, wind) offers a promising route toward sustainable nitrogen conversion. The variable nature of renewable electricity complements the dynamic operation of pulsed systems, enabling responsive load balancing and improved energy utilization. Advances in power electronics and smart control systems can further optimize pulse profiles in real time to match energy supply conditions. Moreover, decentralized, renewable‐powered electrolyzers could enable localized production of nitrogen‐based chemicals in off‐grid or agricultural settings.^[^ ^118^, ^119^, ^120^ ^]^ However, ensuring system stability and performance under fluctuating inputs remains a key research priority.

## 6 Summary

**Figure 7:**
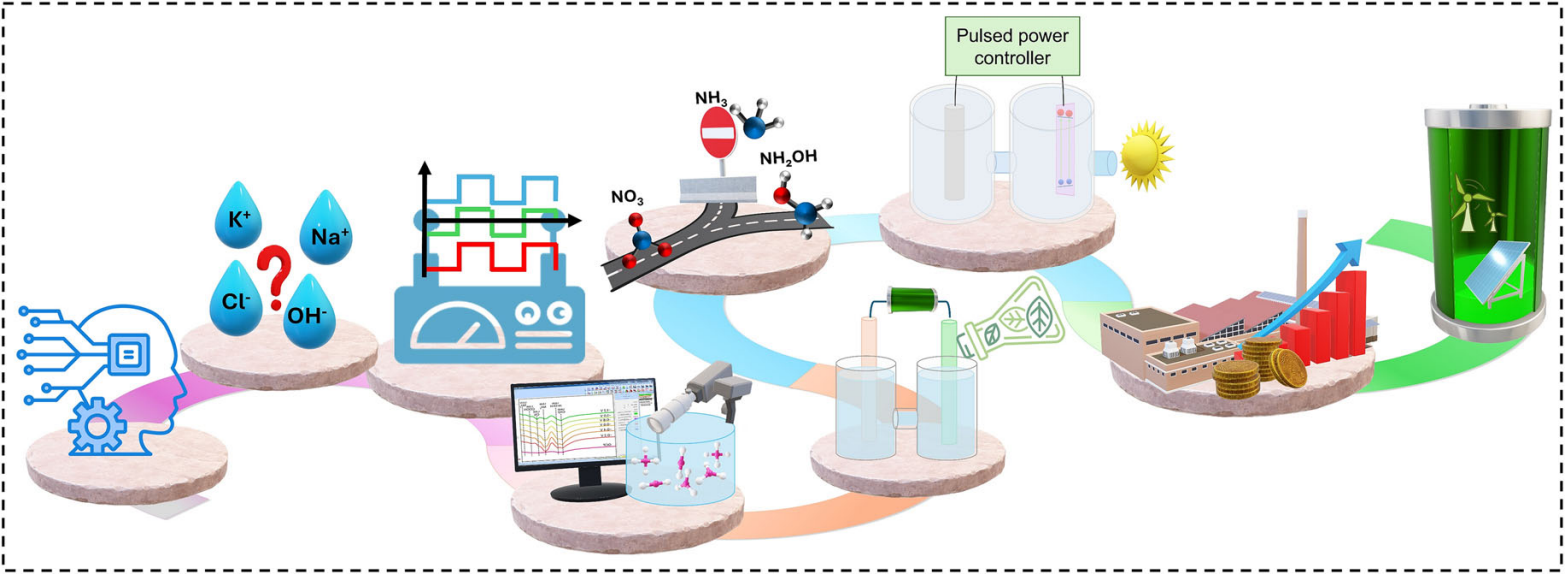
Illustration of outlook for established and emerging directions in pulsed electrolysis.

This review highlights pulsed electrolysis as a promising strategy to overcome key limitations of conventional potentiostatic methods for nitrogen species activation, leveraging rational electrocatalyst modulation and microenvironment engineering. While this research field is still in its infancy, integrating data‐driven optimization of pulsing parameters with advanced experimental and theoretical approaches holds significant potential for uncovering fundamental structure–activity relationships under dynamic conditions. The analysis of emerging research directions underscores the transformative potential of pulsed electrolysis in enabling sustainable, controllable, and scalable nitrogen‐based chemical synthesis (Figure 7).

## Conflict of Interests

The authors declare no conflict of interest.

## Data Availability

Data sharing is not applicable to this article as no new data were created or analyzed in this study.

## References

[1] B. Gu , X. Zhang , S. K. Lam , Y. Yu , H. J. M. van Grinsven , S. Zhang , X. Wang , B. L. Bodirsky , S. Wang , J. Duan , C. Ren , L. Bouwman , W. Vries , J. Xu , M. Sutton , D. Chen , Nature 2023, 613, 77–84, 10.1038/s41586-022-05481-8. 36600068 PMC9842502

[2] H. Xu , Y. Ma , J. Chen , W. X. Zhang , J. Yang , Chem. Soc. Rev. 2022, 51, 2710–2758, 10.1039/D1CS00857A. 35274646

[3] P. H. van Langevelde , I. Katsounaros , M. T. M. Koper , Joule 2021, 5, 290–294, 10.1016/j.joule.2020.12.025.

[4] H. Zhang , K. Fang , J. Yang , H. Chen , J. Ning , H. Wang , Y. Hu , Coord. Chem. Rev. 2024, 506, 215723, 10.1016/j.ccr.2024.215723.

[5] E. Murphy , Y. Liu , I. Matanovic , M. Rüscher , Y. Huang , A. Ly , S. Guo , W. Zang , X. Yan , A. Martini , J. Timoshenko , B. R. Cuenya , I. V. Zenyuk , X. Pan , E. D. Spoerke , P. Atanassov , Nat. Commun. 2023, 14, 4554, 10.1038/s41467-023-40174-4. 37507382 PMC10382506

[6] H. Qian , Z. Yuan , N. Chen , X. Zhu , S. Huang , C. Lu , K. Liu , F. Zhou , P. Smith , H. Tian , X. Qu , J. Zou , S. Liu , Z. Song , W. Zhang , S. Wang , Z. Liu , G. Li , Z. Shang , Y. Ding , K. Groenigen , Y. Jiang , Nat. Commun. 2025, 16, 2775, 10.1038/s41467-025-58090-0. 40113803 PMC11926090

[7] B. Aryal , R. Gurung , A. F. Camargo , G. Fongaro , H. Treichel , B. Mainali , M. J. Angove , H. H. Ngo , W. Guo , S. R. Puadel , Environ. Pollut. 2022, 314, 120272, 10.1016/j.envpol.2022.120272. 36167167

[8] M. Guo , Y. Zhang , C. Guo , Y. Yu , Angew. Chem. Int. Ed. 2025, 64, e202509053. 10.1002/anie.202509053 40377256

[9] S. O. Simonetti , S. B. Beil , S. R. Waldvogel , ACS Electrochem. 2025, 6, 805–818.

[10] M. A. Nawaz , R. Blay‐Roger , M. Saif , F. Meng , J. González‐Arias , B. Miao , L. F. Bobadilla , T. Ramirez‐Reina , J. A. Odriozola , ACS Catal. 2023, 13, 14415–14453, 10.1021/acscatal.3c02410.

[11] J. Lim , C. A. Fernández , S. W. Lee , M. C. Hatzell , ACS Energy Lett. 2021, 6, 3676–3685, 10.1021/acsenergylett.1c01614.

[12] G. F. Costa , M. Escudero‐Escribano , JACS Au 2025, 5, 1538–1548, 10.1021/jacsau.5c00065. 40313822 PMC12042039

[13] M. Jiang , M. Zhu , M. Wang , Y. He , X. Luo , C. Wu , L. Zhang , Z. Jin , ACS Nano 2023, 17, 3209–3224, 10.1021/acsnano.2c11046. 36786415

[14] J. John , D. R. MacFarlane , A. N. Simonov , Nat. Catal. 2023, 6, 1125–1130, 10.1038/s41929-023-01060-w.

[15] Y. Xiong , Y. Wang , J. Zhou , F. Liu , F. Hao , Z. Fan , Adv. Mater. 2024, 36, 2304021, 10.1002/adma.202304021. 37294062

[16] C. Lv , L. Zhong , H. Liu , Z. Fang , C. Yan , M. Chen , Y. Kong , C. Lee , D. Liu , S. Li , J. Liu , L. Song , G. Chen , Q. Yan , G. Yu , Nat. Sustain. 2021, 4, 868–876, 10.1038/s41893-021-00741-3.

[17] Y. Liu , X. Yu , X. Li , X. Liu , C. Ye , T. Ling , X. Wang , Z. Zhu , J. Shan , ACS Nano 2024, 18, 23894–23911, 10.1021/acsnano.4c06516. 39160683

[18] D. Chen , J. Liu , J. Shen , Y. Zhang , H. Shao , C. Chen , S. Wang , Adv. Energy Mater. 2024, 14, 2303820, 10.1002/aenm.202303820.

[19] K. Huang , K. Tang , M. Wang , Y. Wang , T. Jiang , M. Wu , Adv. Funct. Mater. 2024, 34, 2315324, 10.1002/adfm.202315324.

[20] T. Hou , T. Shan , H. Rong , J. Zhang , ChemSusChem 2025, 18, e202402331. 39676306 10.1002/cssc.202402331

[21] S. Meng , Y. Ling , M. Yang , X. Zhao , A. I. Osman , A. H. Al‐Muhtaseb , D. W. Rooney , P. S. Yap , J. Environ. Chem. Eng. 2023, 11, 109418, 10.1016/j.jece.2023.109418.

[22] F. Shafiq , L. Yang , W. Zhu , Phys. Chem. Chem. Phys. 2024, 26, 11208–11216, 10.1039/D4CP00659C. 38564180

[23] Z. Masaud , G. Liu , L. E. Roseng , K. Wang , Chem. Eng. J. 2023, 475, 145882, 10.1016/j.cej.2023.145882.

[24] F. Liu , R. Zhou , C. Zhang , Z. Wu , H. Ren , H. Y. Ng , Chem. Eng. J. 2024, 479, 147588, 10.1016/j.cej.2023.147588.

[25] T. Liu , J. Wang , X. Yang , M. Gong , J. Energy Chem. 2021, 59, 69–82, 10.1016/j.jechem.2020.10.027.

[26] R. C. DiDomenico , T. Hanrath , ACS Energy Lett. 2022, 7, 292–299, 10.1021/acsenergylett.1c02166.

[27] F. Rocha , J. Proost , Int. J. Hydrogen Energy 2021, 46, 28925–28935, 10.1016/j.ijhydene.2020.11.232.

[28] S. Zhang , X. Cao , B. Wang , J. Wei , L. Zhou , J. Han , J. Yun , Int. J. Hydrogen Energy 2025, 109, 684–693, 10.1016/j.ijhydene.2025.02.158.

[29] W. Chen , Y. He , Y. Zou , S. Wang , Natl. Sci. Open 2024, 3, 20240047, 10.1360/nso/20240047.

[30] T. Ito , J. Raj , T. Zhang , S. Roy , J. Wu , EES Catal. 2024, 2, 997–1005, 10.1039/D4EY00039K.

[31] H. Huang , R. Bi , J. Cui , M.‐M. Hu , L. Tian , X. Yang , L. Zhang , J. Energy Chem. 2021, 59, 405–418, 10.1016/j.jechem.2020.11.027.

[32] M. Garedew , C. H. Lam , L. Petitjean , S. Huang , B. Song , F. Lin , J. E. Jackson , C. M. Saffron , P. T. Anastas , Green Chem. 2021, 23, 2868–2899, 10.1039/D0GC04127K.

[33] M. Guo , L. Fang , L. Zhang , M. Li , M. Cong , X. Guan , C. Shi , C. Gu , X. Liu , Y. Wang , X. Ding , Angew. Chem. Int. Ed. 2023, 62, e202217635, 10.1002/anie.202217635. 36744701

[34] Y. Huang , C. He , C. Cheng , S. Han , M. He , Y. Wang , N. Meng , B. Zhang , Q. Lu , Y. Yu , Nat. Commun. 2023, 14, 7368, 10.1038/s41467-023-43179-1. 37963900 PMC10645723

[35] Z. Wang , Y. Liu , S. Liu , Y. Cao , S. Qiu , F. Deng , Catalysts 2023, 13, 1410, 10.3390/catal13111410.

[36] M. He , Y. Wu , R. Li , Y. Wang , C. Liu , B. Zhang , Nat. Commun. 2023, 14, 5088, 10.1038/s41467-023-40892-9. 37607922 PMC10444869

[37] J. M. McEnaney , S. J. Blair , A. C. Nielander , J. A. Schwalbe , D. M. Koshy , M. Cargnello , T. F. Jaramillo , ACS Sustain. Chem. Eng. 2020, 8, 2672–2681, 10.1021/acssuschemeng.9b05983.

[38] E. R. Corson , J. Guo , W. A. Tarpeh , J. Electrochem. Soc. 2024, 171, 046503, 10.1149/1945-7111/ad3a22.

[39] F. Dou , F. Guo , B. Li , K. Zhang , N. Graham , W. Yu , J. Hazard. Mater. 2024, 472, 134522, 10.1016/j.jhazmat.2024.134522. 38714057

[40] Y. Fu , S. Wang , Y. Wang , P. Wei , J. Shao , T. Liu , G. Wang , X. Bao , Angew. Chem. Int. Ed. 2023, 62, e202303327. 10.1002/anie.202303327 37119055

[41] Y. Wang , W. Zhou , R. Jia , Y. Yu , B. Zhang , Angew. Chem. Int. Ed. 2020, 59, 5350–5354 10.1002/anie.201915992 31965695

[42] Q. Wu , X. Fan , B. Shan , Y. Liu , Nat. Commun. 2025, 16, 3479, 10.1038/s41467-025-58811-5. 40216792 PMC11992037

[43] J. Zhang , L. Huang , W. W. Tjiu , C. Wu , M. Zhang , S. Bin Dolmanan , S. Wang , M. Wang , S. Xi , Z. Aabdin , Y. Lum , J. Am. Chem. Soc. 2024, 146, 30708–30714, 10.1021/jacs.4c13219. 39440633

[44] L. Bai , F. Franco , J. Timoshenko , C. Rettenmaier , F. Scholten , H. S. Jeon , A. Yoon , M. Rüscher , A. Herzog , F. T. Haase , S. Kühl , S. Chee , A. Bergmann , R. Beatriz , J. Am. Chem. Soc. 2024, 146, 9665–9678, 10.1021/jacs.3c13288. 38557016 PMC11009949

[45] X. Yu , K. Li , F. Li , B. Wang , S. Sun , Y. Tang , Z. Li , K. Zhao , Appl. Surf. Sci. 2025, 686, 162078, 10.1016/j.apsusc.2024.162078.

[46] J. Zhou , F. Pan , Q. Yao , Y. Zhu , H. Ma , J. Niu , J. Xie , Appl. Catal. B Environ. 2022, 317, 121811, 10.1016/j.apcatb.2022.121811.

[47] Y. Xu , Y. Wen , T. Ren , H. Yu , K. Deng , Z. Wang , X. Li , L. Wang , H. Wang , Appl. Catal. B Environ. 2023, 320, 121981, 10.1016/j.apcatb.2022.121981.

[48] X. Feng , J. Liu , Y. Kong , Z. Zhang , Z. Zhang , S. Li , L. Tong , X. Gao , J. Zhang , Adv. Mater. 2024, 36, 2405660, 10.1002/adma.202405660. 38884637

[49] Y. Bai , Z. Fang , K. Jia , X. Jiang , Y. Gao , C. Lin , D. Ma , J. Li , H. Bai , W. Fan , Small 2025, 21, 2408546, 10.1002/smll.202408546. 39676347

[50] Q. Chen , X. An , Q. Liu , X. Wu , L. Xie , J. Zhang , W. Yao , M. S. Hamdy , Q. Kong , X. Sun , Chem. Commun. 2022, 58, 517–520, 10.1039/D1CC06215H. 34908040

[51] F. Liu , Z. Wu , D. Wang , H. Ren , R. Zhou , C. Zhang , Chem. Eng. J. 2025, 507, 160652, 10.1016/j.cej.2025.160652.

[52] Y. Bu , C. Wang , W. Zhang , X. Yang , J. Ding , G. Gao , Angew. Chem. Int. Ed. 2023, 62, e202217337. 10.1002/anie.202217337 37074107

[53] N. Zhou , Z. Wang , N. Zhang , D. Bao , H. Zhong , X. Zhang , ACS Catal. 2023, 13, 7529–7537, 10.1021/acscatal.3c01315.

[54] Y. Bu , W. Yu , Q. Yang , W. Zhang , Q. Sun , W. Wu , P. Cui , C. Wang , G. Gao , Environ. Sci. Technol. 2024, 58, 12708–12718, 10.1021/acs.est.4c02445. 38953681

[55] Z. Wei , S. Yu , C. Li , Catal. Sci. Technol. 14, 2024, 5128–5142, 10.1039/D4CY00726C.

[56] K. W. Jeon , S. Huo , B. I. Espinosa , X. Wang , Top. Catal. 2024, 10.1007/s11244-024-01933-9.

[57] Y. Chu , Y. Cheng , P. Wang , J. Bai , X. Guan , S. Wang , C. Lan , H. Wu , Z. Shi , S. Zhu , W. Liu , C. Liu , M. Xiao , W. Xing , Sci. China Chem. 2025, 68, 1541–1549, 10.1007/s11426-024-2359-9.

[58] S. Xiang , X. Zhang , Acta Metall. Sin. English Lett. 2020, 33, 281–289, 10.1007/s40195-019-00941-z.

[59] F. Lv , M. Sun , Y. Hu , J. Xu , W. Huang , N. Han , B. Huang , Y. Li , Energy Environ. Sci. 2022, 16, 201–209, 10.1039/D2EE02647C.

[60] W. Chen , L. Zhang , L. Xu , Y. He , H. Pang , S. Wang , Y. Zou , Nat. Commun. 2024, 15, 2420, 10.1038/s41467-024-46752-4. 38499522 PMC10948758

[61] Y. Bu , W. Yu , W. Zhang , C. Wang , J. Ding , G. Gao , Nano Lett. 2024, 24, 2812–2820, 10.1021/acs.nanolett.3c04920. 38396345

[62] J. Wang , H. Y. Tan , M. Y. Qi , J. Y. Li , Z. R. Tang , N. T. Suen , Y. J. Xu , H. M. Chen , Chem. Soc. Rev. 2023, 52, 5013–5050, 10.1039/D2CS00441K. 37431250

[63] J. Zhang , Z. Zhang , T. Chen , J. Zhang , Y. Zhang , Nanomaterials 2025, 15, 648, 10.3390/nano15090648. 40358265 PMC12074274

[64] M. Heßelmann , D. Felder , W. Plischka , S. Nabi , J. Linkhorst , M. Wessling , R. Keller , Angew. Chem. Int. Ed. 2024, 63, e202406924. 10.1002/anie.202406924 38884252

[65] K. Ye , T. W. Jiang , H. D. Jung , P. Shen , S. M. Jang , Z. Weng , S. Back , W. Bin Cai , K. Jiang , Nat. Commun. 2024, 15, 9781, 10.1038/s41467-024-54122-3. 39532852 PMC11557597

[66] P. Li , R. Li , Y. Liu , M. Xie , Z. Jin , G. Yu , J. Am. Chem. Soc. 2023, 145, 6471–6479, 10.1021/jacs.3c00334. 36897656

[67] R. Zhao , Q. Yan , L. Lu , L. Yu , H. Chen , T. Yan , L. Liu , J. Xi , ACS Catal. 14, 2024, 17046–17054, 10.1021/acscatal.4c03782.

[68] Q. Yang , Y. Bu , S. Pu , L. Chu , W. Huang , X. Zhu , C. Liu , G. Fang , P. Cui , D. Zhou , Y. Wang , Angew. Chem. Int. Ed. 2024, 63, e202400428, 10.1002/anie.202400428. 38291811

[69] X. Zhu , J. Huang , M. Eikerling , Acc. Chem. Res. 2024, 57, 2080–2092, 10.1021/acs.accounts.4c00234. 39031075 PMC11308366

[70] A. Paradelo Rodríguez , G. Mul , B. T. Mei , ACS Eng. Au 2025, 5, 27–35. 39990648 10.1021/acsengineeringau.4c00035 PMC11843601

[71] R. Boppella , M. Ahmadi , B. M. Arndt , D. R. Lustig , M. Nazemi , ACS Catal. 2024, 14, 18223–18236, 10.1021/acscatal.4c05225.

[72] K. Zhang , G. Liu , Q. Wang , X. Huo , X. Zou , M. Tang , X. Zhang , L. An , Adv. Sci. 2025, e07720, 10.1002/advs.202507720. PMC12561188 40719047

[73] Z. Li , Z. Shi , Y. Ou , L. Zhong , C. Yan , C. Zhang , K. Song , H. Liu , D. Liu , P. Song , H. Liu , D. Liu , P. Song , C. Yin , Z. Qi , L. Song , C. Lv , Angew. Chem. Int. Ed. 2025, e202510287. 10.1002/anie.202510287 40708400

[74] Q. Hu , W. Zhou , S. Qi , Q. Huo , X. Li , M. Lv , X. Chen , C. Feng , J. Yu , X. Chai , H. Yang , C. He , Nat. Sustain. 2024, 7, 442–451, 10.1038/s41893-024-01302-0.

[75] C. S. Gerke , M. Klenk , P. Zapol , V. S. Thoi , ACS Catal. 2023, 13, 14540–14547, 10.1021/acscatal.3c03027.

[76] W. Qiu , S. Qin , Y. Li , N. Cao , W. Cui , Z. Zhang , Z. Zhuang , D. Wang , Y. Zhang , Angew. Chem. Int. Ed. 2024, 63, e202402684, 10.1002/anie.202402684. 38597346

[77] J. C. Vantourout , H. N. Miras , A. Isidro‐Llobet , S. Sproules , A. J. B. Watson , J. Am. Chem. Soc. 2017, 139, 4769–4779, 10.1021/jacs.6b12800. 28266843

[78] M. J. West , J. W. B. Fyfe , J. C. Vantourout , A. J. B. Watson , Chem. Rev. 2019, 119, 12491–12523, 10.1021/acs.chemrev.9b00491. 31756093

[79] S. N. Steinmann , Q. Wang , Z. W. Seh , Mater. Horiz. 2023, 10, 393–406, 10.1039/D2MH01279K. 36541226

[80] J. Feng , Y. Ji , Y. Li , J. Mater. Chem. A 2023, 11, 14195–14203, 10.1039/D3TA01883K.

[81] J. Liu , S. Wang , Y. Tian , H. Guo , X. Chen , W. Lei , Y. Yu , C. Wang , Angew. Chem. Int. Ed. 2025, 64, e202414314, 10.1002/anie.202414314. 39264257

[82] A. T. Gardini , U. Raucci , M. Parrinello , Nat. Commun. 2025, 16, 2475, 10.1038/s41467-025-57688-8. 40074737 PMC11903671

[83] D. Singh , S. Razzaq , S. Faridi , K. S. Exner , Mater. Today 2025, 86, 575–579, 10.1016/j.mattod.2025.03.016.

[84] Q. Gao , H. S. Pillai , Y. Huang , S. Liu , Q. Mu , X. Han , Z. Yan , H. Zhou , Q. He , H. Xin , H. Zhu , Nat. Commun. 2022, 13, 2338, 10.1038/s41467-022-29926-w. 35487883 PMC9054787

[85] Y. Hu , B. Hu , H. Lan , J. Gong , R. Hu , D. Wang , W.‐D. Zhang , M. Yan , Q. Wang , Y. Liu , H. Xia , M. Yao , M. Du , ACS Appl. Mater. Interfaces 2025, 17, 6161–6174, 10.1021/acsami.4c14956. 39731547

[86] J. Liu , X. Guo , T. Frauenheim , Y. Gu , L. Kou , Adv. Funct. Mater. 2024, 34, 2313420, 10.1002/adfm.202313420.

[87] Q. Wu , Z. J. Xu , Angew. Chem. Int. Ed. 2025, 64, e202505022. 10.1002/anie.202505022 40247668

[88] X. Mao , X. Bai , G. Wu , Q. Qin , A. P. O'Mullane , Y. Jiao , A. Du , J. Am. Chem. Soc. 2024, 146, 18743–18752, 10.1021/jacs.4c06629. 38916520

[89] W. Wen , S. Fang , Y. Zhou , Y. Zhao , P. Li , X. Y. Yu , Angew. Chem. Int. Ed. 2024, 63, e202408382, 10.1002/anie.202408382. 38806407

[90] W. Liu , M. Xia , C. Zhao , B. Chong , J. Chen , H. Li , H. Ou , G. Yang , Nat. Commun. 2024, 15, 3524, 10.1038/s41467-024-47765-9. 38664388 PMC11045753

[91] S.‐J. Qian , H. Cao , X.‐M. Lv , J. Li , Y.‐G. Wang , J. Am. Chem. Soc. 2025, 147, 21032–21040, 10.1021/jacs.5c05728. 40459522

[92] H. Jiang , G.‐F. Chen , O. Savateev , J. Xue , L.‐X. Ding , Z. Liang , M. Antonietti , H. Wang , Angew. Chem. Int. Ed. 2023, 62, e202218717. 10.1002/anie.202218717 36728627

[93] S. Lu , G. Lin , H. Yan , Y. Li , T. Qi , Y. Li , S. Liang , L. Jiang , ACS Catal. 2024, 14, 14887–14894, 10.1021/acscatal.4c05292.

[94] Y. Wang , W. Zhou , R. Jia , Y. Yu , B. Zhang , Angew. Chem. Int. Ed. 2020, 59, 5350–5354, 10.1002/anie.201915992. 31965695

[95] X. Feng , J. Liu , Y. Kong , Z. Zhang , Z. Zhang , S. Li , L. Tong , X. Gao , J. Zhang , Adv. Mater. 2024, 36, 2311434. 10.1002/adma.202405660 38884637

[96] R. Gao , J. Zhang , G. Fan , X. Wang , F. Ding , Y. Guo , C. Han , Y. Gao , A. Shen , J. Ding , L. Wu , X. Gu , Angew. Chem. Int. Ed. 2025, 64, e202505948, 10.1002/anie.202505948. 40192546

[97] C. Yang , F. Yue , T. Wei , X. Li , W. Zhu , C. Wang , Y. Zhen , F. Fu , Y. Liang , J. Energy Chem. 2025, 102, 73–83, 10.1016/j.jechem.2024.10.023.

[98] Y. Wang , A. Xu , Z. Wang , L. Huang , J. Li , F. Li , J. Wicks , M. Luo , D. H. Nam , C. S. Tan , Y. Ding , J. Wu , Y. Lum , C.T. Dinh , D. Sinton , G. Zheng , E. H. Sargent , J. Am. Chem. Soc. 2020, 142, 5702–5708, 10.1021/jacs.9b13347. 32118414

[99] J. S. Barroso‐Martínez , M. Escudero‐Escribano , ChemCatChem 2025, 17, e00352, 10.1002/cctc.202500352.

[100] J. Zhang , T. Quast , B. Eid , Y. T. Chen , R. Zerdoumi , S. Dieckhöfer , J. R. C. Junqueira , S. Seisel , W. Schuhmann , Nat. Commun. 2024, 15, 8583, 10.1038/s41467-024-52780-x. 39362855 PMC11450097

[101] C. Nickel , D. L. Troglauer , Z. Dallos , D. Abid , K. Sowa , M. O. Cichocka , U. Kolb , B. Mashtakov , B. F. Mohazzab , S. Han , L. Prädel , L. Ci , D. Li , X. Lin , M. Hua , R. Liu , D. Gao , Angew. Chem. Int. Ed. 2025, 64, e202424074. 10.1002/anie.202424074 PMC12258672 39907043

[102] B. F. Mohazzab , K. Torabi , D. Gao , Sustain. Energy Fuels 2024, 8, 5620–5637, 10.1039/D4SE01034E.

[103] G. R. Xu , M. Batmunkh , S. Donne , H. Jin , J. X. Jiang , Y. Chen , T. Ma , J. Mater. Chem. A 2019, 7, 25433–25440, 10.1039/C9TA10267A.

[104] Q. Qin , T. Heil , J. Schmidt , M. Schmallegger , G. Gescheidt , M. Antonietti , M. Oschatz , ACS Appl. Energy Mater. 2019, 2, 8359–8365, 10.1021/acsaem.9b01852.

[105] J. Wu , X. Cheng , Y. Tong , Z. Yu , C. Lin , N. Zhang , L. Chen , P. Chen , ACS Catal. 2024, 14, 18095–18106, 10.1021/acscatal.4c05434.

[106] C. Li , H. Li , B. Zhang , H. Li , Y. Wang , X. Wang , P. Das , Y. Li , X. Wu , Y. Li , Y. Cui , J. Xiao , Z. Wu , Angew. Chem. Int. Ed. 2024, 63, e202411542, 10.1002/anie.202411542. 39132837

[107] X. Wang , J. Wang , H. Hu , C. Yin , L.‐Y. Chang , Y. Zhu , J. Wang , M. Yang , Adv. Mater. 2025, 37, 2504505, 10.1002/adma.202504505. 40304534

[108] J. Wang , H. T. D. Bui , H. Hu , S. Kong , X. Wang , H. Zhu , J. Ma , J. Xu , Y. Liu , L. Liu , W. Chen , H. Bi , M. Yang , F. Huang , T. Brinck , J. Wang , Adv. Mater. 2025, 37, 2418451, 10.1002/adma.202418451. 39981855 PMC11983258

[109] S. Li , P. Ma , C. Gao , L. Liu , X. Wang , M. Shakouri , R. Chernikov , K. Wang , D. Liu , R. Ma , J. Wang , Energy Environ. Sci. 2022, 15, 3004–3014, 10.1039/D2EE00461E.

[110] L. Xiao , W. Dai , S. Mou , X. Wang , Q. Cheng , F. Dong , Energy Environ. Sci. 2023, 16, 2696–2704, 10.1039/D3EE00635B.

[111] L. Zhang , Y. Cai , Y. Li , C. Sun , Y. Xiao , Y. Yang , D. Chen , D. Xiao , C. F. Lee , Y. Wang , S. Feng , H. Wang , Y. Shao , T. Chan , H. Ishii , N. Hiraoka , X. Wang , J. Luo , L. Han , Energy Environ. Sci. 2025, 18, 2804–2816, 10.1039/D4EE04382K.

[112] X. Cheng , Z. Xie , S. Zha , Q. Xu , S. Ci , Z. Wen , J. Mater. Chem. A 2025, 13, 13286–13294, 10.1039/D5TA00172B.

[113] A. Chaturvedi , S. Gaber , S. Kaur , K. C. Ranjeesh , T. C. Nagaiah , D. Shetty , ACS Energy Lett. 2024, 9, 2484–2491, 10.1021/acsenergylett.4c00707.

[114] R. Yang , Y. Wang , H. Li , J. Zhou , Z. Gao , C. Liu , B. Zhang , Angew. Chem. Int. Ed.. 2024, 63, e202317167 10.1002/anie.202317167 38323917

[115] J. Zhou , S. Han , R. Yang , T. Li , W. Li , Y. Wang , Y. Yu , B. Zhang , Angew. Chem. Int. Ed. 2023, 62, e202305184. 10.1002/anie.202305184 37129145

[116] D. H. Kim , S. Ringe , H. Kim , S. Kim , B. Kim , G. Bae , H. S. Oh , F. Jaouen , W. Kim , H. Kim , C. Choi , Nat. Commun. 2021, 12, 1856, 10.1038/s41467-021-22147-7. 33767159 PMC7994811

[117] C. Guo , M. Guo , Y. Zhang , S. Han , Y. Yu , J. Am. Chem. Soc. 2025, 147, 14869–14877, 10.1021/jacs.5c04863. 40247764

[118] Y. Tang , Z. Jiang , Y. Yuan , L. Xu , C. Jin , B. Chen , Z. Lin , J. Zao , J. Du , X. Zhang , X. Gao , Y. Liang , Nat. Commun. 2024, 15, 9800, 10.1038/s41467-024-54204-2. 39532869 PMC11557954

[119] J. Wu , L. Xu , Z. Kong , K. Gu , Y. Lu , X. Wu , Y. Zou , S. Wang , Angew. Chem. Int. Ed. 2023, 62, e202311196, 10.1002/anie.202311196. 37721394

[120] J. E. Kim , J. H. Jang , K. M. Lee , M. Balamurugan , Y. I. Jo , M. Y. Lee , S. Choi , S. W. Im , K. T. Nam , Angew. Chem. Int. Ed. 2021, 60, 21943–21951, 10.1002/anie.202108352. 34324785

[121] M. Li , Y. Wu , B.‐H. Zhao , C. Cheng , J. Zhao , C. Liu , B. Zhang , Nat. Catal. 2023, 6, 906–915, 10.1038/s41929-023-01012-4.

[122] Z. Zhu , Y. Jiang , L. Xu , Q. An , T. T. T. Nga , J. Chen , Y. Fan , Q. Liu , C. L. Dong , S. Wang , Y. Zou , Adv. Mater. 2025, 37, 2409864, 10.1002/adma.202409864. 39668465

[123] G. Wu , Z. Ma , T. Heil , L. Zhang , W. Hu , G. Wu , W. He , L. Dai , Y. Huang , Q. Qin , Adv. Mater. 2025, 37, 2418233, 10.1002/adma.202418233. 39801163

[124] Y. Wu , J. Zhao , C. Wang , T. Li , B. H. Zhao , Z. Song , C. Liu , B. Zhang , Nat. Commun. 2023, 14, 3057, 10.1038/s41467-023-38888-6. 37236928 PMC10219941

[125] W. Chen , Y. Wu , Y. Jiang , G. Yang , Y. Li , L. Xu , M. Yang , B. Wu , Y. Pan , Y. Xu , Q. Liu , C. Chen , F. Peng , S. Wang , Y. Zou , J. Am. Chem. Soc. 2024, 146, 6294–6306, 10.1021/jacs.3c14687. 38377334

[126] R. Xiang , S. Wang , P. Liao , F. Xie , J. Kang , S. Li , J. Xian , L. Guo , G. Li , Angew. Chem. Int. Ed. 2023, 62, e202312239. 10.1002/anie.202312239 37728507

[127] Y. Wu , W. Chen , Y. Jiang , Y. Xu , B. Zhou , L. Xu , C. Xie , M. Yang , M. Qiu , D. Wang , Q. Liu , Q. Liu , S. Wang , Y. Zou , Angew. Chem. Int. Ed. 2023, 62, e202305491, 10.1002/anie.202305491. 37232096

[128] J. Sharp , A. Ciotti , H. Andrews , S. R. Udayasurian , M. García‐Melchor , T. Li , ACS Catal. 2024, 14, 3287–3297, 10.1021/acscatal.3c05388. 38449527 PMC10913030

[129] C. Liu , Y. Gao , B. Zhang , Nat. Synth. 2024, 3, 794–796, 10.1038/s44160-024-00550-4.

[130] J. Ding , Y. Lyu , H. Zhou , B. Johannessen , X. Zhang , J. Zheng , S. P. Jiang , S. Wang , Appl. Catal. B Environ. Energy 2024, 345, 123735, 10.1016/j.apcatb.2024.123735.

[131] X. Chang , T. Wang , P. Yang , G. Zhang , J. Gong , Adv. Mater. 2019, 31, 1804710, 10.1002/adma.201804710. 30537099

[132] M. Li , Q. Shi , Z. Li , M. Xu , S. Yu , Y. Wang , S.‐M. Xu , H. Duan , Angew. Chem. Int. Ed. 2024, 63, e202406515, 10.1002/anie.202406515. 38803131

[133] H. E. Kim , J. Kim , E. C. Ra , H. Zhang , Y. J. Jang , J. S. Lee , Angew. Chem. Int. Ed. 2022, 61, e202204117, 10.1002/anie.202204117. 35384205

[134] Y. Cao , S. Yuan , L. Meng , Y. Wang , Y. Hai , S. Su , W. Ding , Z. Liu , X. Li , M. Luo , ACS Sustain. Chem. Eng. 2023, 11, 7965–7985, 10.1021/acssuschemeng.3c01084.

[135] B. Izelaar , M. Ramdin , A. Vlierboom , M. Pérez‐Fortes , D. van der Slikke , A. Sajeev Kumar , W. de Jong , F. M. Mulder , R. Kortlever , Energy Environ. Sci. 17, 2024, 7983–7998, 10.1039/D4EE03299C. 39398319 PMC11462118

[136] Q. Zhang , W. Ye , W. Chen , W. Zeng , Y. Xu , B. Liang , Q. Wang , S. Wu , X. Dong , Y. Li , X. Ren , H. Cao , D. Zhang , X. Han , S. Ye , J. Liu , Q. Zhang , Angew. Chem. Int. Ed. 2025, 64, e202504815, 10.1002/anie.202504815. 40457899

[137] X. Lu , Z. C. Yao , X. Ma , Z. Q. Shi , L. Ding , J. Fu , Z. H. Lyu , Z. Jiang , S. Q. Wang , J. Yang , X. Chang , B. Xu , J. Hu , J. Am. Chem. Soc. 2025, 147, 19342–19352, 10.1021/jacs.5c05466. 40390374

[138] S. Garcia‐Segura , M. Lanzarini‐Lopes , K. Hristovski , P. Westerhoff , Appl. Catal. B Environ. 2018, 236, 546–568, 10.1016/j.apcatb.2018.05.041.

[139] G. E. Dima , A. C. A. de Vooys , M. T. M. Koper , J. Electroanal. Chem. 2003, 554–555, 15–23, 10.1016/S0022-0728(02)01443-2.

[140] L. Wu , L. Zhang , J. Feng , S. Jia , R. Wang , X. Song , X. Ma , Q. Zhu , X. Kang , Q. Qian , X. Sun , B. Han , Chem 11, 2025, 102591, 10.1016/j.chempr.2025.102591.

[141] H. Xu , Y. Ma , J. Chen , W. Zhang , J. Yang , Chem. Soc. Rev. 2022, 51, 2710–2758, 10.1039/D1CS00857A. 35274646

[142] Z. Wang , S. Liu , M. Wang , L. Zhang , Y. Jiang , T. Qian , J. Xiong , C. Yang , C. Yan , ACS Catal. 2023, 13, 9125–9135, 10.1021/acscatal.3c01821.

[143] L. Xu , X. Ma , L. Wu , X. Tan , X. Song , Q. Zhu , C. Chen , Q. Qian , Z. Liu , X. Sun , S. Liu , B. Han , Angew. Chem. Int. Ed. 2022, 61, e202210375, 10.1002/anie.202210375. 35876024

[144] H. Zhou , Z. Chen , W. Meng , S. Yang , J. Environ. Chem. Eng. 2024, 12, 112892, 10.1016/j.jece.2024.112892.

[145] R. Zhao , Q. Yan , H. Lin , L. Yu , L. Liu , J. Xi , Chem Catal. 2025, 5, 101465.

[146] B. Min , Q. Gao , Z. Yan , X. Han , K. Hosmer , A. Campbell , H. Zhu , Ind. Eng. Chem. Res. 2021, 60, 14635–14650, 10.1021/acs.iecr.1c03072.

